# A genetic risk score for CAD, psychological stress, and their interaction as predictors of CAD, fatal MI, non-fatal MI and cardiovascular death

**DOI:** 10.1371/journal.pone.0176029

**Published:** 2017-04-20

**Authors:** Thomas Svensson, Mariusz Kitlinski, Gunnar Engström, Olle Melander

**Affiliations:** 1 Department of Clinical Sciences, Lund University, Malmö, Sweden; 2 Department of Global Health Policy, Graduate School of Medicine, The University of Tokyo, 7-3-1 Hongo, Bunkyo-ku, Tokyo Japan; 3 Department of Neuropsychiatry, Keio University School of Medicine, 35 Shinanomachi, Shinjuku-ku, Tokyo, Japan; 4 Department of Cardiology, Skåne University Hospital, Malmö, Sweden; 5 Department of Internal Medicine, Skåne University Hospital, Malmö, Sweden; Universitatsklinikum Hamburg-Eppendorf, GERMANY

## Abstract

**Background:**

Psychological stress is an independent risk factor for cardiovascular disease (CVD), but the mechanism by which stress is associated with CVD is not entirely understood. Although genetic factors are implied in both stress responsivity and cardiovascular reactivity, no studies to date have investigated their interactions with stress for cardiovascular end points. The objective was to elucidate the association and interactions between a genetic risk score (GRS), individual genetic variants and stress for three cardiovascular end points: coronary artery disease (CAD), fatal myocardial infarction (MI), non-fatal MI, and cardiovascular death.

**Methods and findings:**

18,559 participants from the Malmö Diet Cancer Study, a population-based prospective study, were included in the analyses. Cox proportional hazards regression models were used and adjusted for a large number of known predictors of cardiovascular end points. Mean follow-up time in years was 14.6 (CAD; n = 1938), 14.8 (fatal MI; n = 436), 14.8 (non-fatal MI; n = 1108), and 15.1 (cardiovascular death; n = 1071) respectively. GRS was significantly associated with increased risks of CAD (top quartile hazard ratio [HR], 1.72; 95% confidence interval [CI], 1.51–1.96), fatal MI (top quartile HR, 1.62; 95%CI, 1.23–2.15), non-fatal MI (top quartile HR, 1.55; 95%CI, 1.31–1.84), and cardiovascular death (top quartile HR, 1.29; 95%CI, 1.08–1.53). Stress was not independently associated with any end point and did not interact with GRS. Four individual genetic variants interacted unfavorably with stress for end points with mortality outcomes.

**Conclusion:**

A GRS composed of 50 SNPs and predictive of CAD was found for the first time to also strongly predict fatal MI, non-fatal MI and cardiovascular death. A stress-sensitive component of the GRS was isolated on the basis of individual genetic variants that interacted unfavorably with stress.

## Introduction

Cardiovascular disease (CVD) is the leading cause of death in the world[1] and accounts for 48% of deaths from noncommunicable diseases[2]. In addition to conventional risk factors (i.e. hypertension, smoking, diabetes, family history, and dyslipidemia), psychological stress is considered an important and potentially modifiable risk factor for CVD[3, 4]. Stress is associated with increased risk of stroke and coronary heart disease (CHD)[5], and the effect of severe global stress with regards to ischemic heart disease (IHD) is comparable to that of hypertension and abdominal obesity[6] with risk gradients of stress on heart disease comparable to those of cholesterol[7]. However, the association between stress and CHD cannot entirely be explained by its mediating effect on traditional risk factors[8]. In fact, stress is an independent risk factor for IHD[6].

Stress is associated with atherosclerosis in a number of ways; acute or chronic stress affects responses related to insulin sensitivity[9], inflammatory processes[10], endothelial dysfunction[11], and blood coagulation[12]. However, susceptibility to stress-mediated cardiovascular events differs between individuals[13]. An individual’s stress reactivity seems to be determined by genetic factors[14], and twin studies propose the existence of certain genes which have an effect on sympathovagal cardiac control, heart rate and blood pressure in rest and stress[15]. This suggests genotypic variations to stress responsivity and stress-induced cardiovascular reactivity.

In recent years, Genome Wide Association Studies (GWAS) have found numerous genetic loci associated with coronary artery disease (CAD) risk at genome wide significance levels[16]. In addition to their independent association with the risk of a cardiovascular event[17], many of these loci are associated with conventional risk factors such as blood lipid concentrations and blood pressure[16]. Despite the rapid emergence and identification of new CAD risk/susceptibility loci, no studies to date have simultaneously investigated psychological stress and its interaction with, and the additive effects of genetic factors in relation to cardiovascular end points. Considering the observed inter-individual differences in stress-mediated cardiovascular events and the strong association between stress and CVD, it is quite possible that stress could modify any inherent genetic risk for CVD[18]. Prospective cohort studies on stress and CHD are called for[4] and the incorporation of stress processes into cardiovascular pathophysiological research is considered the major challenge over the next decade of research[8].

Consequently, the primary aim of this study was to investigate the main effects of, and interactions between a known CHD-susceptibility genetic risk score (GRS)[19] and psychological stress for coronary artery disease (CAD), fatal myocardial infarction (MI), non-fatal MI and cardiovascular death. The secondary aim was to investigate the interaction between individual genetic variants and stress on the four outcomes. We hypothesize that the main effects of, as well as the interactions between GRS and psychological stress will be significantly associated with increased risks of coronary events and of cardiovascular death.

## Methods

The Malmö Diet and Cancer Study is a population-based prospective study in the city of Malmö, Sweden. Between the years 1991–1996, men and women aged 45–73 years were selected at random and recruited for a baseline examination. The study has been described in detail elsewhere[20]. In brief, anthropometric data were taken together with blood samples, and participants were asked to fill in a questionnaire that included items on heredity, socioeconomic variables, and lifestyle factors.

At baseline, 30,447 individuals were identified in the study population. We excluded those with a history of CAD (n = 773) and participants for whom we did not have complete information on psychological stress (n = 5480) or genotyping (n = 5634), leaving us with 18,559 individuals included in the analyses. We did not exclude individuals with a history of CVD from analyses on cardiovascular death for two principal reasons: 1) the 50-variant GRS is associated with CAD, not CVD, and 2) to maintain a uniform population throughout all analyses in the study.

The MDC study was approved by the ethics committee at Lund University, and all participants provided written informed consent.

### Genetic risk score

The first independent variable of interest was a 50-variant GRS associated with CHD which has been described in detail elsewhere[19]. In brief, each of the 50 SNPs included in the GRS is associated with CAD at genome wide significance levels[16, 21–24]. For each allele, the number of carried risk alleles (0/1/2) was multiplied with the natural logarithm transformed literature-based risk estimate for that risk allele[16, 21–24]. The resulting GRS was divided into quartiles where the lowest GRS (Quartile 1) was considered the reference category.

### Genotyping

A multiplex method combining polymerase chain reaction, allele-specific oligonucleotide ligation assays, and hybridization to oligonucleotides coupled to Luminex 100TM xMAP microspheres (Luminex, Austin, TX) was used for the genotyping of MDC participants.[25]

### Psychological stress

Psychological stress was the second variable of interest in the present study. A categorical stress variable was constructed from 11 questions measuring job strain using items in the validated Swedish version[26] of Karasek’s[27] and Theorell’s[28] Demand-Control Model, and one question assessing non-work-related stress. The job strain questionnaire has been used in cardiovascular research[29], and single questions to assess the association between perceived mental stress and cardiovascular end points have been used previously in large prospective studies[5]. Cronbach’s alpha for the 12 items assessing stress in the present study was 0.69.

The Psychological Demands (PD; five items) and the Decision Latitude (DL; six items) subscales of the job strain questionnaire were assessed using a 4-point Likert scale. Scores of each subscale were summarized without weighting and job strain was calculated by dividing PD with DL and the results dichotomized at the median to allow for a ‘low strain and a ‘high strain’ group. Non-work-related stress was assessed using one question: ‘*Have you lately suffered from stress or mental pressure because of problems or demands not related to your work*?’ Answers of ‘yes’ and ‘no’ represented high stress and low stress levels respectively.

The dichotomized variables job strain and non-work related stress were subsequently combined to create a categorical variable representing low, intermediate or high psychological stress. Low psychological stress was assigned to individuals who belonged to the low strain and low stress group, intermediate stress to those with low strain and high stress or high strain and low stress, and high stress was assigned to those with high strain and high stress.

### End points

The study had four primary end points: 1) time to first occurrence of a coronary artery disease event (CAD) defined as a first fatal or non-fatal myocardial infarction, coronary artery bypass graft (CABG) or percutaneous coronary intervention (PCI), 2) time to first occurrence of fatal myocardial infarction (MI), 3) time to first occurrence of non-fatal MI, and 4) time to first occurrence of death due to cardiovascular disease.

All events were identified through linkage of a 10-digit national personal identification number with four registries validated for classification of outcomes as described in detail elsewhere[30–32]: the Swedish National Discharge Registry, the Swedish National Cause of Death Registry, the Swedish Coronary Angiography and Angioplasty Registry, and the Stroke in Malmö Registry. CABG and PCI were classified using the national classification of surgical procedures operation codes (KKÅ or Op6): 3065, 3066, 3068, 3080, 3092, 3105, 3127, 3158 for CABG, and FNG02 and FNG05 for PCI. CE was defined according to the International Classification of Diseases, ninth (ICD-9) and tenth (ICD-10) revisions with fatal or non-fatal MI or death due to CHD corresponding to codes 410, 412, and 414 (ICD-9), and I21-I23 and, I25 (ICD-10). Fatal MI was defined as fatal MI or fatal IHD but where death occurred within 28 days of event, non-fatal MI was defined as MI or IHD without death within 28 days of event, and CVD was defined as codes 390–451 (ICD-9) and I00-I99 (ICD-10). Participants were followed from starting point until December 31, 2010, with person-years calculated from starting point to the date of event, loss to follow-up, or end of follow-up period, whichever came first.

### Statistical analyses

The association between categories of GRS and stress with baseline characteristics was determined using the Chi-square test and one-way analysis of variance for categorical and continuous variables respectively. Cox proportional hazards models were used to estimate the relative risk for the association between GRS, stress, and each of the three end points. For each end point separate multivariable models were created to show main effects of the two independent variables as well as the p-value for their interaction. Effect modification was determined by using the Likelihood ratio test; the multivariable models including an interaction term between GRS and stress were compared to those without this term. No gender-specific stratification was done as there were no significant interactions between gender and any of the main independent variables.

The minimally adjusted models were adjusted for gender and age (continuous) at starting point. The multivariable models were further adjusted for education (elementary school or higher than elementary school), socioeconomic index (SEI), smoking (never-, past- and current smoker), drinking (none, 1–15.2, and ≥15.2 g ethanol/day), prevalent diabetes mellitus, Body Mass Index (BMI) in kg/m^2^ (continuous), hypertension, and use of lipid lowering medication (yes/no).

Hypertension was defined as systolic blood pressure ≥140, diastolic blood pressure ≥90 or use of antihypertensive medication. SEI was categorized according to the Swedish socioeconomic classification[33] (manual worker, low and intermediate level non-manual worker, higher level non-manual worker, other (self-employed incl. farmers), and unemployed). Prevalent diabetes mellitus was defined as self-reported physician diagnosed diabetes, use of antidiabetic medication, fasting blood glucose ≥6.1mmol/l, or belonging to local or national diabetes registries. Missing data were addressed through the construction of dummy variables.

Each SNP was checked for interactions with stress against the four end points in separate multivariable Cox proportional hazards models.

All statistical analyses were performed using SAS (SAS software version 9.3; SAS institute, Inc., Gary, NC). The significance level was set as P<0.05.

## Results

Among subjects free from CAD at baseline, mean follow-up time for analyses of incident CAD, fatal MI, non-fatal MI and cardiovascular death was 14.6, 14.8, 14.8, and 15.1 years, respectively, with 1938 incident CAD events, 436 fatal MIs, 1108 non-fatal MIs, and 1071 cardiovascular deaths during this time. There was a significant difference between quartiles of GRS only in age and alcohol consumption (Table 1). When considering the population according to psychological stress, the largest proportion of individuals considered their overall stress to be intermediate followed by low and high levels of stress. With the exception of BMI and prevalent diabetes mellitus, there were significant differences between groups of stress with regards to all baseline characteristics.

**Table 1:** Baseline characteristics according to Genetic Risk Score (GRS) Quartiles and psychological stress.

	GRS Quartiles					Stress			
Variable	Q1	Q2	Q3	Q4	P-value*	Low	Intermediate	High	P-value*
**Number of individuals**	4640	4640	4641	4638		7234	8227	3098	
**Proportion of total population (%)**	25.0	25.0	25.0	25.0		39.0	44.3	16.7	
**Men (%)**	38.4	38.5	38.0	37.8	n.s.	45.2	35.9	27.9	<0.001
**Age [mean (years ± s.d.)]**	58.3 ± 7.9	58.1 ± 8.0	58.1 ± 7.9	57.9 ± 7.9	0.020	59.0 ± 7.9	58.3 ± 8.0	55.5 ± 7.2	<0.001
**Education (%)**					n.s.				<0.001
Primary school or less	40.8	41.0	40.4	40.4		38.8	44.4	35.0	
Higher than primary school	58.9	58.7	59.4	58.4		61.0	55.3	64.7	
Missing information	0.2	0.3	0.2	0.2		0.2	0.2	0.3	
**Socioeconomic Index (%)**					n.s.				<0.001
Manual worker	35.3	33.1	35.5	35.0		28.9	39.9	34.7	
Lower and intermediate non-manual worker	40.5	42.2	39.8	39.8		44.5	37.7	39.2	
Higher non-manual worker	7.6	8.2	7.9	8.8		9.2	7.5	7.1	
Other (self-employed and farmers)	10.8	10.6	11.5	10.8		13.4	9.0	10.1	
Unemployed	5.7	5.7	5.0	5.2		3.6	5.7	8.7	
Missing information	0.1	0.2	0.3	0.3		0.3	0.2	0.2	
**Smoking status (%)**					n.s.				<0.001
Never	37.7	36.0	38.5	38.5		38.3	38.4	34.4	
Past	34.0	34.8	33.5	33.5		35.8	33.2	31.7	
Current	28.4	29.2	28.0	28.0		25.9	28.5	33.9	
Missing information	0	0	0	0		0	0	0	
**Alcohol consumption (%)**					0.011				<0.001
None	16.2	16.1	15.7	16.3		13.6	16.9	19.6	
1–15.2 g ethanol /day	57.6	57.3	57.4	57.4		57.6	58.3	54.5	
≥ 15.2 g ethanol / day	25.0	25.3	24.7	24.8		27.8	23.3	22.7	
Missing information	1.3	1.3	2.3	1.5		0.9	1.5	3.2	
**BMI [mean (kg/m2 ± s.d.)]**	25.7 ± 4.0	25.7 ± 4.0	25.8 ± 4.0	25.8 ± 4.0	n.s.	25.7 ± 3.8	25.9 ± 4.1	25.8 ± 4.3	n.s.
**Prevalent Diabetes Mellitus (%)**	4.3	4.1	4.4	4.2	n.s.	4.0	4.6	3.8	n.s.
**Hypertension (%)**	39.7	39.9	39.6	41.6	n.s.	41.3	40.4	36.9	<0.001
**Lipid lowering medication (%)**	2.1	2.2	2.6	2.2	n.s.	2.6	2.4	1.4	<0.001

GRS = Genetic risk score; n.s. = non-significant

*Chi-square test for categorical variables, ANOVA for continuous variables.

GRS Quartiles were constructed from a continuous 50 SNP GRS of which all SNPs are associated with CAD at genome wide significance levels.

### CAD

In the multivariable model, main effects for GRS remained significant for the second (hazard ratio [HR], 1.24; 95% confidence interval [CI], 1.08–1.42), third (HR, 1.43; 95% CI, 1.25–1.64) and fourth (HR, 1.72; 95% CI, 1.51–1.96) quartiles (Table 2).

**Table 2:** Hazard Ratios (HR) and Confidence Intervals (CI) for the main effects of genetic risk score (GRS) and stress on incidence of coronary artery disease (CAD), fatal myocardial infarction (MI), non-fatal MI, and cardiovascular death.

	GRS Quartiles					Stress			
	Q1	Q2	Q3	Q4	P for trend	Low	Intermediate	High	P for trend
**CAD**									
No. (Events)	4640 (370)	4640 (462)	4641 (507)	4638 (599)		7234 (817)	8227 (864)	3098 (257)	
Minimal model* HR (95% CI)	Reference	**1.27** **(1.11–1.46)**	**1.44** **(1.26–1.65)**	**1.74** **(1.53–1.98)**		Reference	1.08 (0.98–1.19)	1.11 (0.96–1.28)	
P value	-	<0.001	<0.001	<0.001		-	n.s.	n.s.	
Multivariable model^†^ HR (95% CI)	Reference	**1.24** **(1.08–1.42)**	**1.43** **(1.25–1.64)**	**1.72** **(1.51–1.96)**	**<0.001**	Reference	0.99 (0.90–1.09)	0.99 (0.86–1.14)	n.s.
P value	-	0.002	<0.001	<0.001		-	n.s.	n.s.	
**Fatal MI**									
No. (Events)	4640 (81)	4640 (113)	4641 (116)	4638 (126)		7234 (173)	8227 (203)	3098 (60)	
Minimal model* HR (95% CI)	Reference	**1.42** **(1.06–1.88)**	**1.51** **(1.14–2.01)**	**1.69** **(1.28–2.24)**		Reference	**1.24** **(1.01–1.52)**	**1.43** **(1.06–1.93)**	
P value	-	0.017	0.004	<0.001		-	0.042	0.019	
Multivariable model^†^ HR (95% CI)	Reference	**1.35** **(1.02–1.80)**	**1.49** **(1.12–1.98)**	**1.62** **(1.23–2.15)**	**<0.001**	Reference	1.09 (0.88–1.34)	1.22 (0.90–1.65)	n.s.
P value	-	0.039	0.006	<0.001		-	n.s.	n.s.	
**Non-fatal MI**									
No. (Events)	4640 (227)	4640 (253)	4641 (291)	4638 (337)		7234 (470)	8227 (490)	3098 (148)	
Minimal model* HR (95% CI)	Reference	1.13 (0.94–1.35)	**1.34** **(1.12–1.59)**	**1.57** **(1.33–1.86)**		Reference	1.05 (0.92–1.19)	1.07 (0.89–1.30)	
P value	-	n.s.	0.001	<0.001		-	n.s.	n.s.	
Multivariable model^†^ HR (95% CI)	Reference	1.09 (0.91–1.31)	**1.34** **(1.12–1.59)**	**1.55** **(1.31–1.84)**	**<0.001**	Reference	0.96 (0.85–1.09)	0.97 (0.80–1.17)	n.s.
P value	-	n.s.	0.001	<0.001		-	n.s.	n.s.	
**Cardiovascular death**									
No. (Events)	4640 (239)	4640 (246)	4641 (290)	4638 (296)		7234 (448)	8227 (493)	3098 (130)	
Minimal model* HR (95% CI)	Reference	1.03 (0.86–1.23)	**1.27** **(1.07–1.51)**	**1.32** **(1.12–1.57)**		Reference	1.12 (0.99–1.28)	1.21 (1.00–1.48)	
P value	-	n.s.	0.006	0.001		-	n.s.	n.s.	
Multivariable model^†^ HR (95% CI)	Reference	1.00 (0.83–1.19)	**1.26** **(1.06–1.50)**	**1.29** **(1.08–1.53)**	**<0.001**	Reference	1.02 (0.89–1.16)	1.06 (0.87–1.30)	n.s.
P value	-	n.s.	0.009	0.004		-	n.s.	n.s.	

CAD = Coronary artery disease; CI = Confidence Interval; GRS = Genetic risk score; HR = Hazard Ratio; MI = Myocardial infarction; n.s. = non-significant.

*Minimal models are adjusted for gender and age

^†^Multivariable models are adjusted for gender, age, education, socioeconomic index, smoking, drinking, prevalent diabetes mellitus, Body Mass Index, hypertension, and use of lipid lowering medication

There was no significant interaction between GRS and stress for CAD. Individuals in the top GRS quartile were at the highest risk of CAD irrespective of perceived stress level, and neither intermediate or high stress alone were associated with an increased risk of CAD compared to the reference group (low stress, GRS quartile 1) (Table 3).

**Table 3:** Cox proportional hazards regression models for the effect of interaction between psychological stress and quartiles of coronary artery disease (CAD) genetic risk score (GRS) on incident CAD, fatal myocardial infarction (MI), non-fatal MI, and cardiovascular death.

	GRS Quartiles				
End point	Q1	Q2	Q3	Q4	P for interaction^†^
**CAD**					
No. (Events)	4640 (370)	4640 (462)	4641 (507)	4039 (599)	
	HR (95% CI)	HR (95% CI)	HR (95% CI)	HR (95% CI)	
Minimal model^‡^					
Low stress	Reference	1.35*** (1.09–1.67)	1.53*** (1.24–1.89)	1.92*** (1.56–2.35)	
Intermediate stress	1.15 (0.92–1.44)	1.58*** (1.28–1.95)	1.59*** (1.29–1.96)	1.94*** (1.58–2.38)	
High stress	1.38* (1.02–1.88)	1.13 (0.82–1.57)	1.93*** (1.46–2.57)	2.01*** (1.53–2.65)	
Multivariable model^§^					
Low stress	Reference	1.33* (1.07–1.64)	1.53*** (1.24–1.89)	1.87*** (1.53–2.30)	
Intermediate stress	1.05 (0.84–1.31)	1.41** (1.14–1.75)	1.44*** (1.17–1.77)	1.77*** (1.44–2.18)	
High stress	1.26 (0.92–1.71)	0.94 (0.68–1.31)	1.75*** (1.32–2.33)	1.77*** (1.34–2.33)	n.s.
**Fatal MI**					
No. (Events)	4640 (81)	4640 (113)	4641 (116)	4039 (126)	
	HR (95% CI)	HR (95% CI)	HR (95% CI)	HR (95% CI)	
Minimal model^‡^					
Low stress	Reference	1.59 (0.97–2.62)	2.27*** (1.41–3.65)	2.22** (1.38–3.56)	
Intermediate stress	1.65* (1.00–2.72)	2.53*** (1.59–4.04)	1.94** (1.19–3.15)	2.60*** (1.62–4.17)	
High stress	2.41** (1.27–4.58)	2.04* (1.04–3.99)	2.94*** (1.56–5.52)	2.74** (1.46–5.15)	
Multivariable model^§^					
Low stress	Reference	1.55 (0.94–2.56)	2.24*** (1.39–3.61)	2.15** (1.33–3.46)	
Intermediate stress	1.48 (0.90–2.44)	2.16** (1.35–3.46)	1.66* (1.02–2.71)	2.19** (1.36–3.52)	
High stress	2.06* (1.08–3.92)	1.57 (1.80–3.09)	2.59** (1.37–4.88)	2.30* (1.22–4.32)	n.s.
**Non-fatal MI**					
No. (Events)	4640 (227)	4640 (253)	4641 (291)	4638 (337)	
	HR (95% CI)	HR (95% CI)	HR (95% CI)	HR (95% CI)	
Minimal model^‡^					
Low stress	Reference	1.16 (0.88–1.52)	1.24 (0.94–1.62)	1.54** (1.19–1.99)	
Intermediate stress	0.99 (0.75–1.31)	1.23 (0.94–1.61)	1.35* (1.04–1.76)	1.58*** (1.22–2.05)	
High stress	1.11 (0.74–1.66)	0.78 (0.49–1.23)	1.72** (1.20–2.45)	1.74** (1.23–2.46)	
Multivariable model^§^					
Low stress	Reference	1.14 (0.87–1.49)	1.23 (0.93–1.61)	1.50** (1.16–1.94)	
Intermediate stress	0.90 (0.68–1.19)	1.09 (0.83–1.43)	1.24 (0.95–1.62)	1.45** (1.12–1.89)	
High stress	1.01 (0.68–1.52)	0.65 (0.41–1.04)	1.58* (1.10–2.26)	1.54* (1.09–2.19)	n.s.
**Cardiovascular death**					
No. (Events)	4640 (239)	4640 (246)	4641 (290)	4039 (296)	
	HR (95% CI)	HR (95% CI)	HR (95% CI)	HR (95% CI)	
Minimal model^‡^					
Low stress	Reference	0.83 (0.62–1.11)	1.45** (1.11–1.88)	1.33* (1.02–1.72)	
Intermediate stress	1.11 (0.85–1.46)	1.33* (1.02–1.73)	1.24 (0.95–1.62)	1.47** (1.13–1.92)	
High stress	1.18 (0.78–1.78)	1.34 (0.90–1.99)	1.53* (1.03–2.28)	1.52* (1.03–2.23)	
Multivariable model^§^					
Low stress	Reference	0.82 (0.61–1.09)	1.43** (1.10–1.85)	1.28 (0.98–1.66)	
Intermediate stress	1.01 (0.77–1.32)	1.15 (0.88–1.50)	1.11 (0.85–1.45)	1.31* (1.01–1.71)	
High stress	1.03 (0.68–1.56)	1.10 (0.74–1.63)	1.35 (0.90–2.02)	1.29 (0.88–1.91)	n.s.

*p<0.05

**p<0.01

***p<0.001

CAD = Coronary artery disease; CI = Confidence Interval; GRS = Genetic risk score; HR = Hazard Ratio; MI = Myocardial infarction; n.s. = non-significant.

^†^Likelihood ratio test for overall interaction term

^‡^Minimal models are adjusted for gender and age

^§^Multivariable models are adjusted for gender, age, education, socioeconomic index, smoking, drinking, prevalent diabetes mellitus, Body Mass Index, hypertension, and use of lipid lowering medication

### Fatal MI

Following full adjustment, GRS quartiles 2 (HR, 1.35; 95% CI, 1.02–1.80), 3 (HR, 1.49; 95% CI, 1.12–1.98) and 4 (HR, 1.62; 95% CI, 1.23–2.15) remained significant predictors of fatal MI compared to quartile 1.

When compared to individuals who were in the lowest GRS quartile and reported low stress, individuals with the lowest genetic risk but with high stress were at a significantly increased risk of fatal MI (HR, 2.06; 95% CI, 1.08–3.92). The highest risk of fatal MI was seen for individuals with high stress in GRS quartiles 3 (HR, 2.59; 95% CI, 1.37–4.88) and 4 (HR, 2.30; 95% CI, 1.22–4.32), albeit with a non-significant interaction between GRS and stress.

### Non-fatal MI

Compared to quartile 1 and following full adjustment, GRS quartiles 3 (HR, 1.34; 95% CI, 1.12–1.59), and 4 (HR, 1.55; 95% CI, 1.31–1.84) remained significant predictors of non-fatal MI. Stress was not associated with non-fatal MI.

Individuals in GRS quartile 4 had an increased risk of non-fatal MI at low (HR, 1.50; 95% CI, 1.16–1.94), intermediate (HR, 1.45; 95% CI, 1.12–1.89) and high (HR, 1.54; 95% CI, 1.09–2.19) stress levels. Additionally, those in GRS quartile 3 with high stress (HR, 1.58; 95% CI, 1.10–2.26) were at an increased risk of non-fatal MI. The interaction between GRS and stress was, however, non-significant.

### Cardiovascular death

In the multivariable model, those in the third (HR, 1.26; 95% CI, 1.06–1.50) and fourth (HR, 1.29; 95% CI, 1.08–1.53) quartiles of GRS presented with increased risks of cardiovascular death, with stress levels remaining as non-significant positive associations.

The interaction between GRS and stress was non-significant.

### Interactions between individual genetic variants and stress and risk of CAD, fatal MI or cardiovascular death

Of the 50 genetic variants included in our GRS, simple effects indicated a significantly increased risk of CAD, fatal MI, non-fatal MI, and cardiovascular death for nine SNPs, three SNPs, three SNPs, and six SNPs respectively (Tables A1-A2 in S1 File). The simple effects of eight SNPs indicated increased risks of more than one end point: rs2252641 (ZEB2-AC074093.1), and rs11984041 (HDAC9) predicted both CAD and cardiovascular death, rs3217992 (CDKN2BAS) and rs216172 (SMG6) were associated with CAD and fatal MI, rs10455872 (LPA), rs4977574 (CDKN2A), and rs2259816 (HNF1A) were associated with CAD and non-fatal MI, while rs2895811 (HHIPL1) was associated with both fatal MI and cardiovascular death.

There were a total of 14 significant interactions between genetic variants and perceived stress level (intermediate or high stress) for all end points (Table 4). Intermediate stress interacted with four unique SNPs (twice for fatal MI, once for non-fatal MI, and three times for cardiovascular death), whereas high stress interacted with eleven unique SNPs (twice for CAD, twice for fatal MI, five times for non-fatal MI, and five times for cardiovascular death). Of the interactions that were significant for more than one end point, one was with intermediate stress [rs2895811 (HHIPL1)] and three were with high stress levels [rs2259816 (HNF1A), rs2252641 (ZEB2-AC074093.1), and rs2954029 (TRIB1)].

**Table 4:** Cox proportional hazards multivariable models for the significant interactions between individual genetic variants and stress for each of coronary artery disease (CAD), fatal myocardial infarction (MI), non-fatal MI, and cardiovascular death.

	CAD				Fatal MI				Non-fatal MI				Cardiovascular death			
SNP (Gene)	Point estimate	Lower CI	Upper CI	P*	Point estimate	Lower CI	Upper CI	P*	Point estimate	Lower CI	Upper CI	P*	Point estimate	Lower CI	Upper CI	P*
**rs11206510** (PCSK9)																
At low stress	0.94	0.83	1.06	n.s.	0.87	0.66	1.14	n.s.	1.09	0.91	1.29	n.s.	0.97	0.82	1.15	n.s.
At intermediate stress	0.99	0.88	1.11	n.s.	1.16	0.89	1.49	n.s.	1.00	0.85	1.17	n.s.	1.08	0.92	1.27	n.s.
At high stress	0.80	0.64	0.98	n.s.	0.73	0.48	1.12	n.s.	**0.72**	**0.55**	**0.94**	**0.012**	0.82	0.61	1.11	n.s.
**rs12190287** **(TCF21)**																
At low stress	1.10	1.00	1.22	n.s.	1.08	0.87	1.35	n.s.	1.02	0.89	1.16	n.s.	0.98	0.86	1.13	n.s.
At intermediate stress	1.04	0.94	1.14	n.s.	0.96	0.78	1.17	n.s.	1.06	0.93	1.21	n.s.	1.04	0.92	1.19	n.s.
At high stress	1.04	0.87	1.26	n.s.	1.25	0.84	1.85	n.s.	0.98	0.77	1.24	n.s.	**1.43**	**1.09**	**1.88**	**0.017**
**rs1746048** **(CXCL12)**																
At low stress	**1.24**	**1.06**	**1.45**	**0.007**	1.13	0.81	1.58	n.s.	1.21	0.99	1.48	n.s.	1.03	0.84	1.25	n.s.
At intermediate stress	1.14	0.99	1.32	n.s.	1.02	0.76	1.36	n.s.	1.13	0.94	1.37	n.s.	1.06	0.88	1.27	n.s.
At high stress	1.07	0.82	1.39	n.s.	0.84	0.51	1.37	n.s.	0.99	0.71	1.38	n.s.	**0.69**	**0.49**	**0.91**	**0.023**
**rs2259816** **(HNF1A)**																
At low stress	**0.89**	**0.81**	**0.99**	**0.030**	0.85	0.68	1.07	n.s.	**0.85**	**0.74**	**0.98**	**0.021**	0.95	0.83	1.09	n.s.
At intermediate stress	1.03	0.93	1.13	n.s.	0.93	0.76	1.14	n.s.	0.99	0.87	1.13	n.s.	1.00	0.88	1.14	n.s.
At high stress	0.98	0.81	1.17	n.s.	0.59	0.39	0.89	n.s.	1.15	0.91	1.46	**0.030**	**0.67**	**0.51**	**0.88**	**0.025**
**rs4773144** **(COL4A1)**																
At low stress	1.00	0.91	1.11	n.s.	0.97	0.78	1.20	n.s.	0.98	0.86	1.11	n.s.	1.01	0.89	1.16	n.s.
At intermediate stress	1.06	0.96	1.16	n.s.	1.02	0.84	1.25	n.s.	1.08	0.95	1.23	n.s.	0.98	0.86	1.11	n.s.
At high stress	0.99	0.83	1.18	n.s.	**0.60**	**0.40**	**0.88**	**0.036**	1.13	0.90	1.42	n.s.	0.80	0.62	1.03	n.s.
**rs2895811** **(HHIPL1)**																
At low stress	1.05	0.95	1.16	n.s.	**1.32**	**1.07**	**1.64**	**0.010**	0.90	0.79	1.02	n.s.	**1.14**	**1.00**	**1.30**	**0.049**
At intermediate stress	1.06	0.97	1.17	n.s.	0.93	0.76	1.13	**0.016**	**1.19**	**1.05**	**1.34**	**0.002**	0.91	0.80	1.03	**0.014**
At high stress	1.17	0.99	1.40	n.s.	1.50	1.04	2.15	n.s.	1.07	0.85	1.35	n.s.	1.18	0.93	1.51	n.s.
**rs2252641** (ZEB2-AC074093.1)																
At low stress	**1.11**	**1.00**	**1.22**	**0.047**	1.20	0.97	1.50	n.s.	1.08	0.95	1.23	n.s.	**1.15**	**1.00**	**1.31**	**0.044**
At intermediate stress	1.07	0.98	1.18	n.s.	1.05	0.87	1.28	n.s.	1.05	0.93	1.19	n.s.	0.98	0.86	1.11	n.s.
At high stress	0.87	0.73	1.03	**0.018**	0.91	0.64	1.31	n.s.	**0.76**	**0.60**	**0.96**	**0.010**	1.05	0.83	1.33	n.s.
**rs1878406** **(EDNRA)**																
At low stress	**1.15**	**1.00**	**1.32**	n.s.	1.29	0.96	1.73	n.s.	1.09	0.90	1.31	n.s.	**1.32**	**1.10**	**1.57**	**0.003**
At intermediate stress	1.03	0.90	1.18	n.s.	0.81	0.59	1.12	**0.039**	1.10	0.91	1.32	n.s.	1.05	0.87	1.27	n.s.
At high stress	1.04	0.80	1.35	n.s.	0.87	0.48	1.55	n.s.	1.04	0.74	1.46	n.s.	0.87	0.59	1.31	n.s.
**rs273909 (SLC22A4/SLC22A5)**																
At low stress	1.06	0.92	1.21	n.s.	1.15	0.85	1.55	n.s.	0.94	0.78	1.13	n.s.	1.03	0.85	1.24	n.s.
At intermediate stress	1.02	0.88	1.17	n.s.	1.11	0.83	1.48	n.s.	0.98	0.81	1.19	n.s.	1.04	0.86	1.26	n.s.
At high stress	0.75	0.56	1.00	**0.037**	0.82	0.45	1.47	n.s.	0.78	0.54	1.14	n.s.	0.80	0.54	1.20	n.s.
**rs2954029** **(TRIB1)**																
At low stress	1.06	0.96	1.17	n.s.	0.88	0.71	1.08	n.s.	1.08	0.95	1.23	n.s.	**0.85**	**0.74**	**0.97**	**0.015**
At intermediate stress	0.99	0.90	1.09	n.s.	0.99	0.82	1.21	n.s.	0.99	0.87	1.12	n.s.	0.91	0.80	1.03	n.s.
At high stress	1.13	0.95	1.35	n.s.	**1.57**	**1.08**	**2.28**	**0.008**	1.08	0.86	1.36	n.s.	1.23	0.96	1.57	**0.011**
**rs2487928** **(KIAA1462)**																
At low stress	0.96	0.87	1.06	n.s.	0.88	0.71	1.10	n.s.	1.04	0.91	1.18	n.s.	0.99	0.87	1.14	n.s.
At intermediate stress	0.93	0.84	1.02	n.s.	0.72	0.59	0.87	n.s.	1.06	0.93	1.20	n.s.	**0.79**	**0.70**	**0.90**	**0.015**
At high stress	1.03	0.87	1.22	n.s.	0.92	0.65	1.30	n.s.	1.04	0.83	1.31	n.s.	0.93	0.73	1.18	n.s.
**rs11226029** **(PDGFD)**																
At low stress	0.95	0.85	1.06	n.s.	1.02	0.80	1.30	n.s.	0.89	0.77	1.03	n.s.	0.99	0.86	1.15	n.s.
At intermediate stress	0.98	0.88	1.09	n.s.	1.11	0.88	1.39	n.s.	0.97	0.84	1.11	n.s.	1.05	0.91	1.22	n.s.
At high stress	1.17	0.95	1.44	n.s.	0.89	0.59	1.34	n.s.	**1.45**	**1.08**	**1.94**	**0.003**	1.00	0.76	1.33	n.s.
**rs9319428** **(FLT1)**																
At low stress	0.97	0.87	1.08	n.s.	1.01	0.80	1.26	n.s.	0.88	0.77	1.02	n.s.	0.96	0.83	1.10	n.s.
At intermediate stress	1.08	0.97	1.19	n.s.	1.24	1.01	1.52	n.s.	1.06	0.93	1.21	n.s.	**1.23**	**1.08**	**1.40**	**0.012**
At high stress	1.15	0.96	1.38	n.s.	1.20	0.83	1.74	n.s.	1.21	0.95	1.53	**0.028**	0.99	0.77	1.29	n.s.
**rs7173743** **(ADAMTS7)**																
At low stress	1.10	0.99	1.21	n.s.	1.15	0.93	1.44	n.s.	1.03	0.90	1.17	n.s.	0.94	0.82	1.07	n.s.
At intermediate stress	1.02	0.92	1.12	n.s.	0.87	0.72	1.06	n.s.	1.08	0.95	1.22	n.s.	0.92	0.81	1.04	n.s.
At high stress	1.06	0.89	1.27	n.s.	1.67	1.13	2.48	n.s.	0.92	0.73	1.16	n.s.	**1.32**	**1.02**	**1.70**	**0.022**

CAD = Coronary Artery Disease; CI = Confidence Interval; MI = Myocardial Infarction; n.s. = non-significant; SNP = Single nucleotide polymorphism

*P-values are for the interaction between SNP and stress level.

Analyses are adjusted for gender, age, education, socioeconomic index, smoking, drinking, prevalent diabetes mellitus, Body Mass Index, hypertension, and use of lipid lowering medication

### Post-hoc analyses

Post-hoc analyses showed that when constructing a stress-sensitive GRS comprised of the four SNPs [rs12190287 (TCF21), rs2954029 (TRIB1), rs9319428 (FLT1), and rs7173743 (ADAMTS7)] found in this study to interact unfavorably with stress for CAD, fatal MI or cardiovascular death (i.e. end points which include mortality outcomes), there were significant interactions with stress for fatal MI (p = 0.009) and for cardiovascular death (p = 0.004) (Table B in S1 File). Compared to individuals with low stress and in quartile 1 of the stress-sensitive GRS, those with high stress and in quartile 4 of the stress-sensitive GRS had a more than 3-fold increased risk of fatal MI. Moreover, those with high stress but in quartile 1 of the stress-sensitive GRS had a reduced risk of cardiovascular death.

Specific GRS were created on the basis of SNPs clustered according to their possible CAD-related function (Tables C-E in S1 File). Five SNPs had functions related to lipoproteins [rs646776 (SORT1), rs11206510 (PCSK9), rs10455872 (LPA), rs964184 (APOA5), and rs1122608 (LDLR)], five SNPs had functions related to lipids [rs17114036 (PPAP2B), rs3798220 (LPA), rs12413409 (NT5C2), rs2954029 (TRIB1), and rs1412444 (LIPA)], and four SNPs had functions related to inflammation [rs4845625 (IL6R, rs1878406 (EDNRA, rs4252120 (PLG, and rs9319428 (FLT1)]. In the multivariable models, the third (HR, 1.21; 95% CI, 1.02–1.45) and fourth (HR, 1.18; 95% CI, 1.04–1.34) quartiles of the lipoprotein specific GRS, and the fourth quartile (HR, 1.14; 95% CI, 1.01–1.28) of the lipid specific GRS were significantly associated only with the CAD end point. The fourth quartile of the inflammation specific GRS was significantly associated with CAD (HR, 1.23; 95% CI, 1.08–1.39), fatal MI (HR, 1.31; 95% CI, 1.00–1.71), non-fatal MI (HR, 1.21; 95% CI, 1.03–1.43), and cardiovascular death (HR, 1.22; 95% CI, 1.03–1.44).

There was no significant interaction between the lipoprotein-, lipid-, or inflammation specific GRS with stress for any of the end points (data not shown).

## Discussion

This study has shown for the first time that a CAD-susceptibility GRS comprised of 50 genetic variants known to predict CHD[19], is also predictive of fatal MI, non-fatal MI and cardiovascular death. This study has further shown that although individuals with the highest reported stress levels and those in the top GRS quartiles have the highest risk of fatal MI, perceived stress is not independently associated with any of the end points and does not interact with a GRS associated with CHD, fatal MI, non-fatal MI and cardiovascular death. However, perceived stress interacts with a number of individual genetic variants, a finding that may be of importance for the understanding not only of our results, but also for the comprehension of the influence of stress on cardiovascular end points in general, and for the implementation of genetic risk scores in stress-related cardiovascular research.

In this study, participants who belonged to the top quartiles of GRS were at an increased risk of CAD, fatal MI, non-fatal MI, and cardiovascular death. Despite including in our models strong predictors of all end points, there was only minimal change of the significant risks associated with GRS between the minimal and multivariable models. However, the GRS-associated risk patterns differed between health outcomes. Whereas GRS predicted CAD, fatal MI and non-fatal MI in a linear fashion signifying additive effects with each quartile increase, those in the third and fourth quartiles had a nearly 1.3-fold increase of cardiovascular death. Considering the stability of GRS as a predictive variable despite adjustment for important environmental factors, our findings may have little or limited clinical value until a plausible intervention is determined. Moreover, there were differences between GRS specifically with regards to the fatal MI and non-fatal MI outcomes. Whereas individuals in the second quartile of the GRS were at a 1.3-fold significantly increased risk of fatal MI, they did not have an increased risk of non-fatal MI. Given that fatal MI was defined as death within 28 days of event, our results indicate that the 50 SNP CAD GRS at lower quartiles is a marker of worse prognosis following MI. Indeed, CAD GRS is significantly inversely associated with longevity[34], and future studies should investigate the possibility of considering GRS as a prognostic indicator of MI-related survival.

Of the many environmental factors that have been proposed as important in relation to genetic risks of cardiovascular end points, stress is perhaps the one factor where evidence points in the direction of possible genetically determined inter-individual susceptibility[13–15]. In our study, psychological stress was not independently associated with any end points and did not interact with GRS. This overall lack of interaction between GRS and stress could be due to a number of reasons: first, the risk score is in itself such a reliable and powerful variable that it may override any known environmental influence. Second, the perception of stress can be determined by other factors such as education and socioeconomic status which is in accordance with our findings of significant differences in baseline characteristics between stress categories. A third possibility is that we have used a stress-indiscriminate GRS. It is easy to conceive that individuals with existing CAD or atherosclerotic changes would be more sensitive to the effects of chronic stress, and consequently suffer from premature death.

Our primary results and interactions, albeit non-significant, point to a potential effect of stress on CAD and fatal MI that may possibly differ according to the 50 SNP GRS. Secondary results from one-by-one analysis of the 50 SNPs indeed confirm that there exists a stress sensitive component of the GRS. Fourteen genetic variants significantly interacted with increased stress. Only six of these genetic variants have known or suggested functional associations[35, 36] such as LDL metabolism [rs11206510 (PCSK9)], plaque destabilization [rs4773144 (COL4A1_A2)], vasoconstriction and inflammation [rs1878406 (EDNRA)], lipid metabolism [rs2954029 (TRIB1)], angiogenesis and inflammation [rs9319428 (FLT1)], and hypertension [rs12190287 (TCF21)], and they offer little information on how or why these SNPs modify the effects of stress. However, four SNPs interacted with stress in a way which significantly increased the risk of mortality outcomes. Carriers of one of these variants, rs2954029 (TRIB1), who experienced high levels of stress had a 1.6-fold increased risk of fatal MI, and a 1.2-fold increased risk of cardiovascular death compared to those with low stress. Additionally, highly stressed carriers of rs12190287 (TCF21) had a 1.4-fold increased risk of cardiovascular death. Rs2954029 (TRIB1)[37] is associated with sleep regulation and increased expression during sleep deprivation, which in turn is closely correlated with stress[38] whereas rs12190287 (TCF21) is a possible susceptibility locus for hypertension[36]. Both of these variants should be considered in future research on the association between stress and cardiovascular diseases.

In our post-hoc analyses we also created specific GRS based on the SNPs possible CAD-related functions[35, 39, 40]. These phenotype-specific GRS were poorer at predicting outcomes than the original 50 SNP GRS, and they did not interact with stress for any of the end points. Our results indicate that it may be the functional diversity of various SNPs which increases the risk of cardiovascular outcomes rather than the clustering of SNPs of a single phenotype. However, it is important to note that only 21 of our 50 SNPs had a known or suggested CAD-related function. As correctly pointed out by Pilling et al.[34], effect sizes between different GRS represent and explain different proportions of their phenotypes in their association with the respective end points. Therefore, our analyses and subsequent results of clustered SNPs are limited.

These results may nevertheless add credence to the method of investigating and clustering genetic variants according to their interaction with specific environmental exposures, such as stress. Focusing on such stress-sensitive CAD genetic variants may in fact be the key to understanding inter-individual stress responsivity and stress-induced cardiovascular reactivity. It would allow for the creation of stress-sensitive CAD risk scores, thus possibly finding individuals in which environmental interventions through stress modification could be of clinical value for cardiovascular outcomes. Indeed, post-hoc analyses indicate that a stress-sensitive GRS comprised of four unfavorable SNPs can modify the effects of stress on cardiovascular outcomes, a finding which supports genetically determined inter-individual cardiovascular stress reactivity[13–15], and warrants further investigation as well as replication in future studies.

Limitations of this study include random misclassification and a possible underestimation of stress as it was measured at only one point in time. We have looked at psychological stress of a chronic nature, and our results may therefore not be applicable to intense and acute stress. The definition of stress in this study included a component related to occupational stress which may reduce generalizability of our findings to studies with measures of predominantly non-occupational stress. Indeed, although job strain accounts for a proportion of CHD events, the population attributable risk is not as high as those of recognized factors such as smoking, abdominal obesity and physical inactivity[41]. Furthermore, we did not have information on coping strategies or coping behaviors, factors which may reduce the impact of stressors and which are known to be associated with cardiovascular outcomes[42]. Moreover, the results of this study may not be generalizable to other countries. Finally, this study has an increased risk of type I errors given that p-values are not adjusted for multiple comparisons. However, each of the interactions between individual genetic polymorphisms and stress for the respective end points involves a loss of statistical power due to the reduced number of cases at high stress levels of each polymorphism. As such, the use of corrections for multiple comparisons may increase the risk of type II errors. We highly encourage future studies with available genetic information in addition to stress exposure to try to replicate our findings involving the stress-sensitive component of the GRS.

Despite the limitations, this study has several strengths: it was conducted in a large general population cohort with validated definitions of end points and contains genetic information based on a large number of SNPs in addition to a validated component used in the definition of psychological stress. We have also adjusted our models for a large number of variables known to be associated with the respective end points.

## Conclusions

We have for the first time found that a GRS composed of 50 SNPs which is predictive of CAD is also strongly predictive of fatal MI, non-fatal MI, and cardiovascular death. Our results point to a stress-sensitive component of the GRS which could be isolated based on individual genetic variants that interacted unfavorably with stress. Further research on stress-sensitive CAD genetic variants is warranted in order to understand stress-induced cardiovascular reactivity.

## Supporting information

S1 File Supplementary material. (DOCX)
