# Supplementary material for: A genetic risk score for CAD, psychological stress, and their interaction as predictors of CAD, fatal MI, non-fatal MI and cardiovascular death
Source: PLoS One. 2017 Apr 20;12(4):e0176029. doi: 10.1371/journal.pone.0176029 (PMC5398707; doi:10.1371/journal.pone.0176029)
Supplement: S1 File — (DOCX) [file pone.0176029.s001.docx]

**A genetic risk score for CAD, psychological stress, and their interaction as predictors of CAD, fatal MI, non-fatal MI and cardiovascular death**

**Supplementary material**

## Thomas Svensson^1,2,3*^, Mariusz Kitlinski^1,4^, Gunnar Engström^1^, Olle Melander^1,5^

1. Department of Clinical Sciences, Lund University, Malmö, Sweden;
2. Department of Global Health Policy, Graduate School of Medicine, The University of Tokyo,
   7-3-1 Hongo, Bunkyo-ku, Tokyo 113-0033 Japan
3. Department of Neuropsychiatry, Keio University School of Medicine, 35 Shinanomachi, Shinjuku-ku, Tokyo 160-8582, Japan
4. Department of Cardiology, Skåne University Hospital, Malmö, Sweden
5. Department of Internal Medicine, Skåne University Hospital, Malmö, Sweden

**Supplementary material**

**Contents:**

**Supplementary Tables (pages 3-15)**

**SUPPLEMENTARY TABLES**

Table A1. Cox proportional hazards multivariable models for the interactions between individual genetic variants and stress for each of coronary artery disease (CAD), and cardiovascular death

|  |  | **CAD** | | | |  | **Cardiovascular death** | | | |
| --- | --- | --- | --- | --- | --- | --- | --- | --- | --- | --- |
| **SNP (Gene)** |  | **Point estimate** | **Lower CI** | **Upper CI** | **P*** |  | **Point estimate** | **Lower CI** | **Upper CI** | **P*** |
| **rs646776 (SORT1)** |  |  |  |  |  |  |  |  |  |  |
| At low stress |  | 1.03 | 0.92 | 1.16 | n.s. |  | 1.00 | 0.86 | 1.17 | n.s. |
| At intermediate stress |  | 1.20 | 1.07 | 1.35 | n.s. |  | 1.08 | 0.93 | 1.25 | n.s. |
| At high stress |  | 1.20 | 0.97 | 1.48 | n.s. |  | 1.08 | 0.81 | 1.45 | n.s. |
|  |  |  |  |  |  |  |  |  |  |  |
| **rs17114036 (PPAP2B)** |  |  |  |  |  |  |  |  |  |  |
| At low stress |  | 1.12 | 0.93 | 1.34 | n.s. |  | 0.90 | 0.72 | 1.14 | n.s. |
| At intermediate stress |  | 1.34 | 1.11 | 1.62 | n.s. |  | 1.19 | 0.94 | 1.52 | n.s. |
| At high stress |  | 0.89 | 0.65 | 1.21 | n.s. |  | 1.06 | 0.66 | 1.69 | n.s. |
|  |  |  |  |  |  |  |  |  |  |  |
| **rs11206510 (PCSK9)** |  |  |  |  |  |  |  |  |  |  |
| At low stress |  | 0.94 | 0.83 | 1.06 | n.s. |  | 0.97 | 0.82 | 1.15 | n.s. |
| At intermediate stress |  | 0.99 | 0.88 | 1.11 | n.s. |  | 1.08 | 0.92 | 1.27 | n.s. |
| At high stress |  | 0.80 | 0.64 | 0.98 | n.s. |  | 0.82 | 0.61 | 1.11 | n.s. |
|  |  |  |  |  |  |  |  |  |  |  |
| **rs17465637 (MIA3)** |  |  |  |  |  |  |  |  |  |  |
| At low stress |  | 1.06 | 0.95 | 1.19 | n.s. |  | 1.09 | 0.94 | 1.27 | n.s. |
| At intermediate stress |  | 1.06 | 0.95 | 1.19 | n.s. |  | 0.91 | 0.79 | 1.05 | n.s. |
| At high stress |  | 0.99 | 0.81 | 1.20 | n.s. |  | 0.85 | 0.65 | 1.12 | n.s. |
|  |  |  |  |  |  |  |  |  |  |  |
| **rs6725887 (WDR12)** |  |  |  |  |  |  |  |  |  |  |
| At low stress |  | 1.02 | 0.88 | 1.18 | n.s. |  | 0.68 | 0.81 | 1.19 | n.s. |
| At intermediate stress |  | 0.97 | 0.84 | 1.12 | n.s. |  | 0.86 | 0.70 | 1.05 | n.s. |
| At high stress |  | 1.20 | 0.93 | 1.54 | n.s. |  | 1.02 | 0.70 | 1.48 | n.s. |
|  |  |  |  |  |  |  |  |  |  |  |
| **rs9818870 (MRAS)** |  |  |  |  |  |  |  |  |  |  |
| At low stress |  | 1.05 | 0.92 | 1.20 | n.s. |  | 0.91 | 0.75 | 1.10 | n.s. |
| At intermediate stress |  | 1.14 | 1.01 | 1.30 | n.s. |  | 1.02 | 0.85 | 1.21 | n.s. |
| At high stress |  | 1.19 | 0.95 | 1.49 | n.s. |  | 0.84 | 0.60 | 1.19 | n.s. |
|  |  |  |  |  |  |  |  |  |  |  |
| **rs17609940 (ANKS1A)** |  |  |  |  |  |  |  |  |  |  |
| At low stress |  | 1.05 | 0.93 | 1.18 | n.s. |  | 1.11 | 0.94 | 1.30 | n.s. |
| At intermediate stress |  | 0.97 | 0.87 | 1.09 | n.s. |  | 1.02 | 0.87 | 1.20 | n.s. |
| At high stress |  | 1.04 | 0.84 | 1.29 | n.s. |  | 1.06 | 0.79 | 1.44 | n.s. |
|  |  |  |  |  |  |  |  |  |  |  |
| **rs12526453 (PHACTR1)** |  |  |  |  |  |  |  |  |  |  |
| At low stress |  | 0.97 | 0.87 | 1.08 | n.s. |  | 1.10 | 0.96 | 1.27 | n.s. |
| At intermediate stress |  | 0.92 | 0.83 | 1.02 | n.s. |  | 0.98 | 0.86 | 1.12 | n.s. |
| At high stress |  | 0.80 | 0.66 | 0.97 | n.s. |  | 0.89 | 0.69 | 1.15 | n.s. |
|  |  |  |  |  |  |  |  |  |  |  |
| **rs12190287 (TCF21)** |  |  |  |  |  |  |  |  |  |  |
| At low stress |  | 1.10 | 1.00 | 1.22 | n.s. |  | 0.98 | 0.86 | 1.13 | n.s. |
| At intermediate stress |  | 1.04 | 0.94 | 1.14 | n.s. |  | 1.04 | 0.92 | 1.19 | n.s. |
| At high stress |  | 1.04 | 0.87 | 1.26 | n.s. |  | **1.43** | **1.09** | **1.88** | **0.017** |
|  |  |  |  |  |  |  |  |  |  |  |
| **rs3798220 (LPA)** |  |  |  |  |  |  |  |  |  |  |
| At low stress |  | 1.17 | 0.75 | 1.83 | n.s. |  | 1.30 | 0.73 | 2.33 | n.s. |
| At intermediate stress |  | 1.67 | 1.16 | 2.39 | n.s. |  | 1.71 | 1.04 | 2.81 | n.s. |
| At high stress |  | 1.58 | 0.91 | 2.75 | n.s. |  | 1.92 | 0.94 | 3.90 | n.s. |
|  |  |  |  |  |  |  |  |  |  |  |
| **rs10455872 (LPA)** |  |  |  |  |  |  |  |  |  |  |
| At low stress |  | **1.39** | **1.18** | **1.63** | **<0.001** |  | 1.19 | 0.94 | 1.50 | n.s. |
| At intermediate stress |  | 1.16 | 0.98 | 1.36 | n.s. |  | 1.17 | 0.95 | 1.45 | n.s. |
| At high stress |  | 1.28 | 0.93 | 1.77 | n.s. |  | 1.06 | 0.64 | 1.75 | n.s. |
|  |  |  |  |  |  |  |  |  |  |  |
| **rs11556924 (ZC3HC1)** |  |  |  |  |  |  |  |  |  |  |
| At low stress |  | 1.07 | 0.97 | 1.19 | n.s. |  | 1.10 | 0.96 | 1.26 | n.s. |
| At intermediate stress |  | 1.06 | 0.96 | 1.17 | n.s. |  | 0.94 | 0.83 | 1.07 | n.s. |
| At high stress |  | 1.04 | 0.87 | 1.26 | n.s. |  | 1.36 | 1.04 | 1.78 | n.s. |
|  |  |  |  |  |  |  |  |  |  |  |
| **rs4977574 (CDKN2A)** |  |  |  |  |  |  |  |  |  |  |
| At low stress |  | **1.19** | **1.08** | **1.31** | **<0.001** |  | 1.12 | 0.99 | 1.28 | n.s. |
| At intermediate stress |  | 1.12 | 1.02 | 1.23 | n.s. |  | 1.29 | 1.14 | 1.46 | n.s. |
| At high stress |  | 1.14 | 0.96 | 1.35 | n.s. |  | 0.95 | 0.74 | 1.22 | n.s. |
|  |  |  |  |  |  |  |  |  |  |  |
| **rs579459 (ABO)** |  |  |  |  |  |  |  |  |  |  |
| At low stress |  | 1.06 | 0.95 | 1.19 | n.s. |  | 1.04 | 0.89 | 1.22 | n.s. |
| At intermediate stress |  | 1.07 | 0.96 | 1.20 | n.s. |  | 0.96 | 0.82 | 1.11 | n.s. |
| At high stress |  | 0.87 | 0.70 | 1.07 | n.s. |  | 0.84 | 0.62 | 1.14 | n.s. |
|  |  |  |  |  |  |  |  |  |  |  |
|  |  |  |  |  |  |  |  |  |  |  |
|  |  |  |  |  |  |  |  |  |  |  |
| **Table A1 continued** |  |  |  |  |  |  |  |  |  |  |
|  |  |  |  |  |  |  |  |  |  |  |
| **rs1746048 (CXCL12)** |  |  |  |  |  |  |  |  |  |  |
| At low stress |  | **1.24** | **1.06** | **1.45** | **0.007** |  | 1.03 | 0.84 | 1.25 | n.s. |
| At intermediate stress |  | 1.14 | 0.99 | 1.32 | n.s. |  | 1.06 | 0.88 | 1.27 | n.s. |
| At high stress |  | 1.07 | 0.82 | 1.39 | n.s. |  | **0.69** | **0.49** | **0.91** | **0.023** |
| **rs12413409 (CYP17A1)** |  |  |  |  |  |  |  |  |  |  |
| At low stress |  | 0.92 | 0.79 | 1.07 | n.s. |  | 0.97 | 0.79 | 1.20 | n.s. |
| At intermediate stress |  | 1.05 | 0.90 | 1.22 | n.s. |  | 0.95 | 0.78 | 1.16 | n.s. |
| At high stress |  | 0.93 | 0.70 | 1.23 | n.s. |  | 0.94 | 0.63 | 1.39 | n.s. |
|  |  |  |  |  |  |  |  |  |  |  |
| **rs964184 (APOA5)** |  |  |  |  |  |  |  |  |  |  |
| At low stress |  | 1.11 | 0.96 | 1.27 | n.s. |  | 0.98 | 0.80 | 1.19 | n.s. |
| At intermediate stress |  | 0.97 | 0.84 | 1.12 | n.s. |  | 1.00 | 0.83 | 1.21 | n.s. |
| At high stress |  | 1.04 | 0.81 | 1.33 | n.s. |  | 1.31 | 0.96 | 1.80 | n.s. |
|  |  |  |  |  |  |  |  |  |  |  |
| **rs2259816 (HNF1A)** |  |  |  |  |  |  |  |  |  |  |
| At low stress |  | **0.89** | **0.81** | **0.99** | **0.030** |  | 0.95 | 0.83 | 1.09 | n.s. |
| At intermediate stress |  | 1.03 | 0.93 | 1.13 | n.s. |  | 1.00 | 0.88 | 1.14 | n.s. |
| At high stress |  | 0.98 | 0.81 | 1.17 | n.s. |  | **0.67** | **0.51** | **0.88** | **0.025** |
|  |  |  |  |  |  |  |  |  |  |  |
| **rs3184504 (SH2B3)** |  |  |  |  |  |  |  |  |  |  |
| At low stress |  | 1.03 | 0.93 | 1.13 | n.s. |  | 0.99 | 0.87 | 1.13 | n.s. |
| At intermediate stress |  | 1.11 | 1.01 | 1.22 | n.s. |  | 1.09 | 0.96 | 1.24 | n.s. |
| At high stress |  | 1.04 | 0.87 | 1.24 | n.s. |  | 1.01 | 0.79 | 1.29 | n.s. |
|  |  |  |  |  |  |  |  |  |  |  |
| **rs4773144 (COL4A1)** |  |  |  |  |  |  |  |  |  |  |
| At low stress |  | 1.00 | 0.91 | 1.11 | n.s. |  | 1.01 | 0.89 | 1.16 | n.s. |
| At intermediate stress |  | 1.06 | 0.96 | 1.16 | n.s. |  | 0.98 | 0.86 | 1.11 | n.s. |
| At high stress |  | 0.99 | 0.83 | 1.18 | n.s. |  | 0.80 | 0.62 | 1.03 | n.s. |
|  |  |  |  |  |  |  |  |  |  |  |
| **rs2895811 (HHIPL1)** |  |  |  |  |  |  |  |  |  |  |
| At low stress |  | 1.05 | 0.95 | 1.16 | n.s. |  | **1.14** | **1.00** | **1.30** | **0.049** |
| At intermediate stress |  | 1.06 | 0.97 | 1.17 | n.s. |  | 0.91 | 0.80 | 1.03 | **0.014** |
| At high stress |  | 1.17 | 0.99 | 1.40 | n.s. |  | 1.18 | 0.93 | 1.51 | n.s. |
|  |  |  |  |  |  |  |  |  |  |  |
| **rs3825807 (ADAMTS7)** |  |  |  |  |  |  |  |  |  |  |
| At low stress |  | 1.02 | 0.92 | 1.12 | n.s. |  | 0.92 | 0.81 | 1.05 | n.s. |
| At intermediate stress |  | 0.99 | 0.90 | 1.09 | n.s. |  | 0.88 | 0.77 | 0.99 | n.s. |
| At high stress |  | 0.96 | 0.80 | 1.14 | n.s. |  | 1.14 | 0.89 | 1.45 | n.s. |
|  |  |  |  |  |  |  |  |  |  |  |
| **rs12936587 (RASD1)** |  |  |  |  |  |  |  |  |  |  |
| At low stress |  | 1.04 | 0.95 | 1.15 | n.s. |  | 0.99 | 0.87 | 1.12 | n.s. |
| At intermediate stress |  | 1.05 | 0.95 | 1.15 | n.s. |  | 1.09 | 0.96 | 1.23 | n.s. |
| At high stress |  | 1.15 | 0.97 | 1.37 | n.s. |  | 1.24 | 0.97 | 1.57 | n.s. |
|  |  |  |  |  |  |  |  |  |  |  |
| **rs216172 (SMG6)** |  |  |  |  |  |  |  |  |  |  |
| At low stress |  | **1.12** | **1.01** | **1.24** | **0.029** |  | 1.09 | 0.95 | 1.25 | n.s. |
| At intermediate stress |  | 1.05 | 0.95 | 1.15 | n.s. |  | 0.93 | 0.82 | 1.07 | n.s. |
| At high stress |  | 1.00 | 0.83 | 1.19 | n.s. |  | 1.12 | 0.88 | 1.43 | n.s. |
|  |  |  |  |  |  |  |  |  |  |  |
| **rs318090 (UBE2Z)** |  |  |  |  |  |  |  |  |  |  |
| At low stress |  | 0.99 | 0.90 | 1.09 | n.s. |  | 0.99 | 0.87 | 1.13 | n.s. |
| At intermediate stress |  | 1.11 | 1.01 | 1.22 | n.s. |  | 1.04 | 0.92 | 1.18 | n.s. |
| At high stress |  | 1.18 | 0.99 | 1.40 | n.s. |  | 1.19 | 0.93 | 1.53 | n.s. |
|  |  |  |  |  |  |  |  |  |  |  |
| **rs1122608 (LDLR)** |  |  |  |  |  |  |  |  |  |  |
| At low stress |  | 1.01 | 0.90 | 1.14 | n.s. |  | 0.98 | 0.84 | 1.15 | n.s. |
| At intermediate stress |  | 1.04 | 0.93 | 1.17 | n.s. |  | 0.99 | 0.85 | 1.15 | n.s. |
| At high stress |  | 1.00 | 0.82 | 1.23 | n.s. |  | 1.06 | 0.80 | 1.42 | n.s. |
|  |  |  |  |  |  |  |  |  |  |  |
| **rs9982601 (KCNE2)** |  |  |  |  |  |  |  |  |  |  |
| At low stress |  | 1.05 | 0.91 | 1.21 | n.s. |  | 1.06 | 0.88 | 1.29 | n.s. |
| At intermediate stress |  | 1.11 | 0.97 | 1.27 | n.s. |  | 0.95 | 0.79 | 1.15 | n.s. |
| At high stress |  | 1.08 | 0.84 | 1.38 | n.s. |  | 1.15 | 0.82 | 1.60 | n.s. |
|  |  |  |  |  |  |  |  |  |  |  |
| **rs4845625 (IL6R)** |  |  |  |  |  |  |  |  |  |  |
| At low stress |  | 1.04 | 0.95 | 1.15 | n.s. |  | 1.09 | 0.95 | 1.24 | n.s. |
| At intermediate stress |  | 1.14 | 1.04 | 1.26 | n.s. |  | 1.00 | 0.88 | 1.13 | n.s. |
| At high stress |  | 1.00 | 0.84 | 1.20 | n.s. |  | 0.90 | 0.69 | 1.16 | n.s. |
|  |  |  |  |  |  |  |  |  |  |  |
| **rs2028900 (GGCX/VAMP8)** |  |  |  |  |  |  |  |  |  |  |
| At low stress |  | 1.07 | 0.97 | 1.18 | n.s. |  | 0.93 | 0.81 | 1.06 | n.s. |
| At intermediate stress |  | 0.99 | 0.90 | 1.09 | n.s. |  | 0.92 | 0.81 | 1.04 | n.s. |
| At high stress |  | 1.09 | 0.91 | 1.30 | n.s. |  | 1.08 | 0.85 | 1.38 | n.s. |
|  |  |  |  |  |  |  |  |  |  |  |
| **rs4299376 (ABCG8)** |  |  |  |  |  |  |  |  |  |  |
| At low stress |  | 1.06 | 0.95 | 1.18 | n.s. |  | **1.18** | **1.02** | **1.38** | **0.030** |
| At intermediate stress |  | 0.95 | 0.86 | 1.06 | n.s. |  | 1.03 | 0.90 | 1.18 | n.s. |
| At high stress |  | 1.05 | 0.86 | 1.30 | n.s. |  | 1.01 | 0.77 | 1.31 | n.s. |
|  |  |  |  |  |  |  |  |  |  |  |
| **Table A1 continued** |  |  |  |  |  |  |  |  |  |  |
|  |  |  |  |  |  |  |  |  |  |  |
| **rs515135 (APOB)** |  |  |  |  |  |  |  |  |  |  |
| At low stress |  | 0.97 | 0.85 | 1.10 | n.s. |  | 1.04 | 0.87 | 1.24 | n.s. |
| At intermediate stress |  | 1.08 | 0.95 | 1.23 | n.s. |  | 0.95 | 0.81 | 1.13 | n.s. |
| At high stress |  | 0.97 | 0.76 | 1.23 | n.s. |  | 1.05 | 0.74 | 1.48 | n.s. |
|  |  |  |  |  |  |  |  |  |  |  |
| **rs2252641 (ZEB2-AC074093.1)** |  |  |  |  |  |  |  |  |  |  |
| At low stress |  | **1.11** | **1.00** | **1.22** | **0.047** |  | **1.15** | **1.00** | **1.31** | **0.044** |
| At intermediate stress |  | 1.07 | 0.98 | 1.18 | n.s. |  | 0.98 | 0.86 | 1.11 | n.s. |
| At high stress |  | 0.87 | 0.73 | 1.03 | **0.018** |  | 1.05 | 0.83 | 1.33 | n.s. |
|  |  |  |  |  |  |  |  |  |  |  |
| **rs1878406 (EDNRA)** |  |  |  |  |  |  |  |  |  |  |
| At low stress |  | **1.15** | **1.00** | **1.32** | n.s. |  | **1.32** | **1.10** | **1.57** | **0.003** |
| At intermediate stress |  | 1.03 | 0.90 | 1.18 | n.s. |  | 1.05 | 0.87 | 1.27 | n.s. |
| At high stress |  | 1.04 | 0.80 | 1.35 | n.s. |  | 0.87 | 0.59 | 1.31 | n.s. |
|  |  |  |  |  |  |  |  |  |  |  |
| **rs7692387 (GUCY1A3)** |  |  |  |  |  |  |  |  |  |  |
| At low stress |  | 1.08 | 0.95 | 1.22 | n.s. |  | 1.01 | 0.85 | 1.19 | n.s. |
| At intermediate stress |  | 1.08 | 0.96 | 1.22 | n.s. |  | 1.08 | 0.92 | 1.26 | n.s. |
| At high stress |  | 1.01 | 0.80 | 1.26 | n.s. |  | 1.04 | 0.76 | 1.42 | n.s. |
|  |  |  |  |  |  |  |  |  |  |  |
| **rs273909 (SLC22A4/SLC22A5)** |  |  |  |  |  |  |  |  |  |  |
| At low stress |  | 1.06 | 0.92 | 1.21 | n.s. |  | 1.03 | 0.85 | 1.24 | n.s. |
| At intermediate stress |  | 1.02 | 0.88 | 1.17 | n.s. |  | 1.04 | 0.86 | 1.26 | n.s. |
| At high stress |  | 0.75 | 0.56 | 1.00 | **0.037** |  | 0.80 | 0.54 | 1.20 | n.s. |
|  |  |  |  |  |  |  |  |  |  |  |
| **rs10947789 (KCNK5)** |  |  |  |  |  |  |  |  |  |  |
| At low stress |  | 1.09 | 0.97 | 1.22 | n.s. |  | 1.02 | 0.87 | 1.18 | n.s. |
| At intermediate stress |  | 0.98 | 0.88 | 1.09 | n.s. |  | 1.02 | 0.89 | 1.18 | n.s. |
| At high stress |  | 1.15 | 0.94 | 1.41 | n.s. |  | 1.03 | 0.78 | 1.36 | n.s. |
|  |  |  |  |  |  |  |  |  |  |  |
| **rs2048327 (SLC22A3/LPAL2/LPA)** |  |  |  |  |  |  |  |  |  |  |
| At low stress |  | 1.08 | 0.98 | 1.19 | n.s. |  | 1.04 | 0.91 | 1.19 | n.s. |
| At intermediate stress |  | 1.07 | 0.98 | 1.18 | n.s. |  | 1.04 | 0.91 | 1.18 | n.s. |
| At high stress |  | 0.97 | 0.82 | 1.16 | n.s. |  | 0.95 | 0.74 | 1.22 | n.s. |
|  |  |  |  |  |  |  |  |  |  |  |
| **rs4252120 (PLG)** |  |  |  |  |  |  |  |  |  |  |
| At low stress |  | 1.08 | 0.97 | 1.21 | n.s. |  | 1.00 | 0.86 | 1.15 | n.s. |
| At intermediate stress |  | 1.04 | 0.94 | 1.16 | n.s. |  | 1.04 | 0.90 | 1.19 | n.s. |
| At high stress |  | 1.24 | 1.02 | 1.51 | n.s. |  | 1.30 | 0.98 | 1.71 | n.s. |
|  |  |  |  |  |  |  |  |  |  |  |
| **rs11984041 (HDAC9)** |  |  |  |  |  |  |  |  |  |  |
| At low stress |  | **1.25** | **1.06** | **1.46** | **0.007** |  | **1.25** | **1.01** | **1.55** | **0.044** |
| At intermediate stress |  | 1.14 | 0.97 | 1.33 | n.s. |  | 1.04 | 0.83 | 1.30 | n.s. |
| At high stress |  | 1.16 | 0.87 | 1.55 | n.s. |  | 1.14 | 0.76 | 1.71 | n.s. |
|  |  |  |  |  |  |  |  |  |  |  |
| **rs10953541 (BCAP29)** |  |  |  |  |  |  |  |  |  |  |
| At low stress |  | 1.06 | 0.94 | 1.19 | n.s. |  | 0.94 | 0.81 | 1.09 | n.s. |
| At intermediate stress |  | 0.94 | 0.85 | 1.05 | n.s. |  | 1.08 | 0.93 | 1.24 | n.s. |
| At high stress |  | 1.03 | 0.85 | 1.26 | n.s. |  | 1.04 | 0.78 | 1.37 | n.s. |
|  |  |  |  |  |  |  |  |  |  |  |
| **rs2954029 (TRIB1)** |  |  |  |  |  |  |  |  |  |  |
| At low stress |  | 1.06 | 0.96 | 1.17 | n.s. |  | **0.85** | **0.74** | **0.97** | **0.015** |
| At intermediate stress |  | 0.99 | 0.90 | 1.09 | n.s. |  | 0.91 | 0.80 | 1.03 | n.s. |
| At high stress |  | 1.13 | 0.95 | 1.35 | n.s. |  | 1.23 | 0.96 | 1.57 | **0.011** |
|  |  |  |  |  |  |  |  |  |  |  |
| **rs3217992 (CDKN2BAS)** |  |  |  |  |  |  |  |  |  |  |
| At low stress |  | **1.16** | **1.05** | **1.28** | **0.004** |  | 1.13 | 0.99 | 1.29 | n.s. |
| At intermediate stress |  | 1.02 | 0.92 | 1.12 | n.s. |  | 1.10 | 0.96 | 1.25 | n.s. |
| At high stress |  | 1.22 | 1.02 | 1.46 | n.s. |  | 0.91 | 0.70 | 1.19 | n.s. |
|  |  |  |  |  |  |  |  |  |  |  |
| **rs2487928 (KIAA1462)** |  |  |  |  |  |  |  |  |  |  |
| At low stress |  | 0.96 | 0.87 | 1.06 | n.s. |  | 0.99 | 0.87 | 1.14 | n.s. |
| At intermediate stress |  | 0.93 | 0.84 | 1.02 | n.s. |  | **0.79** | **0.70** | **0.90** | **0.015** |
| At high stress |  | 1.03 | 0.87 | 1.22 | n.s. |  | 0.93 | 0.73 | 1.18 | n.s. |
|  |  |  |  |  |  |  |  |  |  |  |
| **rs2047009 (CXCL12)** |  |  |  |  |  |  |  |  |  |  |
| At low stress |  | 1.04 | 0.94 | 1.15 | n.s. |  | 1.06 | 0.93 | 1.21 | n.s. |
| At intermediate stress |  | 1.12 | 1.02 | 1.23 | n.s. |  | 0.94 | 0.83 | 1.06 | n.s. |
| At high stress |  | 1.21 | 1.01 | 1.44 | n.s. |  | 1.03 | 0.81 | 1.31 | n.s. |
|  |  |  |  |  |  |  |  |  |  |  |
| **rs1412444 (LIPA)** |  |  |  |  |  |  |  |  |  |  |
| At low stress |  | 1.07 | 0.96 | 1.19 | n.s. |  | 1.06 | 0.92 | 1.22 | n.s. |
| At intermediate stress |  | 1.04 | 0.94 | 1.16 | n.s. |  | 1.02 | 0.89 | 1.17 | n.s. |
| At high stress |  | 1.10 | 0.92 | 1.31 | n.s. |  | 1.28 | 1.00 | 1.64 | n.s. |
|  |  |  |  |  |  |  |  |  |  |  |
| **rs11226029 (PDGFD)** |  |  |  |  |  |  |  |  |  |  |
| At low stress |  | 0.95 | 0.85 | 1.06 | n.s. |  | 0.99 | 0.86 | 1.15 | n.s. |
| At intermediate stress |  | 0.98 | 0.88 | 1.09 | n.s. |  | 1.05 | 0.91 | 1.22 | n.s. |
| At high stress |  | 1.17 | 0.95 | 1.44 | n.s. |  | 1.00 | 0.76 | 1.33 | n.s. |
|  |  |  |  |  |  |  |  |  |  |  |
| **Table A1 continued** |  |  |  |  |  |  |  |  |  |  |
|  |  |  |  |  |  |  |  |  |  |  |
| **rs9319428 (FLT1)** |  |  |  |  |  |  |  |  |  |  |
| At low stress |  | 0.97 | 0.87 | 1.08 | n.s. |  | 0.96 | 0.83 | 1.10 | n.s. |
| At intermediate stress |  | 1.08 | 0.97 | 1.19 | n.s. |  | **1.23** | **1.08** | **1.40** | **0.012** |
| At high stress |  | 1.15 | 0.96 | 1.38 | n.s. |  | 0.99 | 0.77 | 1.29 | n.s. |
|  |  |  |  |  |  |  |  |  |  |  |
| **rs9515203 (COL4A1/COL4A2)** |  |  |  |  |  |  |  |  |  |  |
| At low stress |  | 1.08 | 0.97 | 1.21 | n.s. |  | 1.03 | 0.89 | 1.20 | n.s. |
| At intermediate stress |  | 1.13 | 1.01 | 1.25 | n.s. |  | 1.07 | 0.93 | 1.23 | n.s. |
| At high stress |  | 0.95 | 0.79 | 1.14 | n.s. |  | 0.91 | 0.70 | 1.17 | n.s. |
|  |  |  |  |  |  |  |  |  |  |  |
| **rs7173743 (ADAMTS7)** |  |  |  |  |  |  |  |  |  |  |
| At low stress |  | 1.10 | 0.99 | 1.21 | n.s. |  | 0.94 | 0.82 | 1.07 | n.s. |
| At intermediate stress |  | 1.02 | 0.92 | 1.12 | n.s. |  | 0.92 | 0.81 | 1.04 | n.s. |
| At high stress |  | 1.06 | 0.89 | 1.27 | n.s. |  | **1.32** | **1.02** | **1.70** | **0.022** |
|  |  |  |  |  |  |  |  |  |  |  |
| **rs17514846 (FURIN/FES)** |  |  |  |  |  |  |  |  |  |  |
| At low stress |  | **1.11** | **1.00** | **1.22** | **0.045** |  | 1.01 | 0.88 | 1.16 | n.s. |
| At intermediate stress |  | 1.03 | 0.94 | 1.14 | n.s. |  | 1.08 | 0.95 | 1.22 | n.s. |
| At high stress |  | 0.98 | 0.82 | 1.17 | n.s. |  | 0.97 | 0.76 | 1.24 | n.s. |

*P-values are for the interaction between SNP and stress level.

CAD=Coronary Artery Disease; CI=Confidence Interval; MI=Myocardial Infarction; n.s.=non-significant; SNP=Single nucleotide polymorphism;

Analyses are adjusted for gender, age, education, socioeconomic index, smoking, drinking, prevalent diabetes mellitus, Body Mass Index, hypertension, and use of lipid lowering medication

Table A2. Cox proportional hazards multivariable models for the interactions between individual genetic variants and stress for each of fatal myocardial infarction (MI), and non-fatal MI

|  |  | **Fatal MI** | | | |  | **Non-fatal MI** | | | |
| --- | --- | --- | --- | --- | --- | --- | --- | --- | --- | --- |
| **SNP (Gene)** |  | **Point estimate** | **Lower CI** | **Upper CI** | **P*** |  | **Point estimate** | **Lower CI** | **Upper CI** | **P*** |
| **rs646776 (SORT1)** |  |  |  |  |  |  |  |  |  |  |
| At low stress |  | 0.93 | 0.73 | 1.20 | n.s. |  | 1.00 | 0.86 | 1.16 | n.s. |
| At intermediate stress |  | 1.31 | 1.02 | 1.67 | n.s. |  | 1.23 | 1.05 | 1.43 | n.s. |
| At high stress |  | 1.19 | 0.77 | 1.84 | n.s. |  | 1.18 | 0.90 | 1.56 | n.s. |
|  |  |  |  |  |  |  |  |  |  |  |
| **rs17114036 (PPAP2B)** |  |  |  |  |  |  |  |  |  |  |
| At low stress |  | 1.17 | 0.78 | 1.75 | n.s. |  | 1.03 | 0.81 | 1.30 | n.s. |
| At intermediate stress |  | 1.58 | 1.03 | 2.42 | n.s. |  | 1.36 | 1.06 | 1.75 | n.s. |
| At high stress |  | 0.92 | 0.47 | 1.78 | n.s. |  | 0.89 | 0.59 | 1.35 | n.s. |
|  |  |  |  |  |  |  |  |  |  |  |
| **rs11206510 (PCSK9)** |  |  |  |  |  |  |  |  |  |  |
| At low stress |  | 0.87 | 0.66 | 1.14 | n.s. |  | 1.09 | 0.91 | 1.29 | n.s. |
| At intermediate stress |  | 1.16 | 0.89 | 1.49 | n.s. |  | 1.00 | 0.85 | 1.17 | n.s. |
| At high stress |  | 0.73 | 0.48 | 1.12 | n.s. |  | **0.72** | **0.55** | **0.94** | **0.012** |
|  |  |  |  |  |  |  |  |  |  |  |
| **rs17465637 (MIA3)** |  |  |  |  |  |  |  |  |  |  |
| At low stress |  | 1.09 | 0.85 | 1.40 | n.s. |  | 0.99 | 0.86 | 1.15 | n.s. |
| At intermediate stress |  | 0.99 | 0.79 | 1.24 | n.s. |  | 1.06 | 0.91 | 1.22 | n.s. |
| At high stress |  | 0.74 | 0.50 | 1.10 | n.s. |  | 1.18 | 0.90 | 1.54 | n.s. |
|  |  |  |  |  |  |  |  |  |  |  |
| **rs6725887 (WDR12)** |  |  |  |  |  |  |  |  |  |  |
| At low stress |  | 0.98 | 0.71 | 1.34 | n.s. |  | 1.02 | 0.84 | 1.23 | n.s. |
| At intermediate stress |  | 0.70 | 0.50 | 0.99 | n.s. |  | 0.94 | 0.78 | 1.14 | n.s. |
| At high stress |  | 1.18 | 0.69 | 2.01 | n.s. |  | 1.16 | 0.83 | 1.62 | n.s. |
|  |  |  |  |  |  |  |  |  |  |  |
| **rs9818870 (MRAS)** |  |  |  |  |  |  |  |  |  |  |
| At low stress |  | 0.99 | 0.74 | 1.34 | n.s. |  | 1.12 | 0.94 | 1.33 | n.s. |
| At intermediate stress |  | 1.25 | 0.97 | 1.61 | n.s. |  | 1.06 | 0.90 | 1.26 | n.s. |
| At high stress |  | 1.01 | 0.63 | 1.62 | n.s. |  | 1.22 | 0.92 | 1.63 | n.s. |
|  |  |  |  |  |  |  |  |  |  |  |
| **rs17609940 (ANKS1A)** |  |  |  |  |  |  |  |  |  |  |
| At low stress |  | 1.09 | 0.84 | 1.43 | n.s. |  | 1.06 | 0.90 | 1.24 | n.s. |
| At intermediate stress |  | 0.98 | 0.77 | 1.24 | n.s. |  | 0.98 | 0.84 | 1.14 | n.s. |
| At high stress |  | 0.88 | 0.58 | 1.33 | n.s. |  | 1.22 | 0.91 | 1.65 | n.s. |
|  |  |  |  |  |  |  |  |  |  |  |
| **rs12526453 (PHACTR1)** |  |  |  |  |  |  |  |  |  |  |
| At low stress |  | 1.13 | 0.90 | 1.42 | n.s. |  | 0.91 | 0.79 | 1.05 | n.s. |
| At intermediate stress |  | 0.90 | 0.72 | 1.11 | n.s. |  | 0.92 | 0.81 | 1.06 | n.s. |
| At high stress |  | 0.77 | 0.52 | 1.13 | n.s. |  | 0.88 | 0.69 | 1.13 | n.s. |
|  |  |  |  |  |  |  |  |  |  |  |
| **rs12190287 (TCF21)** |  |  |  |  |  |  |  |  |  |  |
| At low stress |  | 1.08 | 0.87 | 1.35 | n.s. |  | 1.02 | 0.89 | 1.16 | n.s. |
| At intermediate stress |  | 0.96 | 0.78 | 1.17 | n.s. |  | 1.06 | 0.93 | 1.21 | n.s. |
| At high stress |  | 1.25 | 0.84 | 1.85 | n.s. |  | 0.98 | 0.77 | 1.24 | n.s. |
|  |  |  |  |  |  |  |  |  |  |  |
| **rs3798220 (LPA)** |  |  |  |  |  |  |  |  |  |  |
| At low stress |  | 1.84 | 0.83 | 4.08 | n.s. |  | 0.83 | 0.42 | 1.66 | n.s. |
| At intermediate stress |  | 1.80 | 0.85 | 3.81 | n.s. |  | 1.56 | 0.95 | 2.55 | n.s. |
| At high stress |  | 1.52 | 0.48 | 4.85 | n.s. |  | 1.26 | 0.56 | 2.84 | n.s. |
|  |  |  |  |  |  |  |  |  |  |  |
| **rs10455872 (LPA)** |  |  |  |  |  |  |  |  |  |  |
| At low stress |  | 1.21 | 0.83 | 1.76 | n.s. |  | **1.25** | **1.00** | **1.55** | **0.048** |
| At intermediate stress |  | 1.04 | 0.74 | 1.47 | n.s. |  | 1.24 | 1.00 | 1.54 | n.s. |
| At high stress |  | 0.91 | 0.41 | 2.06 | n.s. |  | 1.48 | 1.00 | 2.19 | n.s. |
|  |  |  |  |  |  |  |  |  |  |  |
| **rs11556924 (ZC3HC1)** |  |  |  |  |  |  |  |  |  |  |
| At low stress |  | 1.10 | 0.89 | 1.38 | n.s. |  | 1.09 | 0.95 | 1.24 | n.s. |
| At intermediate stress |  | 1.03 | 0.84 | 1.27 | n.s. |  | 1.08 | 0.95 | 1.23 | n.s. |
| At high stress |  | 1.26 | 0.85 | 1.86 | n.s. |  | 0.93 | 0.73 | 1.18 | n.s. |
|  |  |  |  |  |  |  |  |  |  |  |
| **rs4977574 (CDKN2A)** |  |  |  |  |  |  |  |  |  |  |
| At low stress |  | 1.13 | 0.92 | 1.40 | n.s. |  | **1.22** | **1.08** | **1.39** | **0.002** |
| At intermediate stress |  | 1.29 | 1.06 | 1.57 | n.s. |  | 1.06 | 0.93 | 1.20 | n.s. |
| At high stress |  | 1.02 | 0.71 | 1.46 | n.s. |  | 1.22 | 0.97 | 1.54 | n.s. |
|  |  |  |  |  |  |  |  |  |  |  |
| **rs579459 (ABO)** |  |  |  |  |  |  |  |  |  |  |
| At low stress |  | 1.06 | 0.82 | 1.36 | n.s. |  | 1.06 | 0.92 | 1.24 | n.s. |
| At intermediate stress |  | 1.11 | 0.88 | 1.40 | n.s. |  | 1.08 | 0.93 | 1.25 | n.s. |
| At high stress |  | 0.85 | 0.55 | 1.31 | n.s. |  | 0.80 | 0.60 | 1.07 | n.s. |
|  |  |  |  |  |  |  |  |  |  |  |
| **rs1746048 (CXCL12)** |  |  |  |  |  |  |  |  |  |  |
| At low stress |  | 1.13 | 0.81 | 1.58 | n.s. |  | 1.21 | 0.99 | 1.48 | n.s. |
| At intermediate stress |  | 1.02 | 0.76 | 1.36 | n.s. |  | 1.13 | 0.94 | 1.37 | n.s. |
| At high stress |  | 0.84 | 0.51 | 1.37 | n.s. |  | 0.99 | 0.71 | 1.38 | n.s. |
|  |  |  |  |  |  |  |  |  |  |  |
|  |  |  |  |  |  |  |  |  |  |  |
| **Table A2 continued** |  |  |  |  |  |  |  |  |  |  |
|  |  |  |  |  |  |  |  |  |  |  |
| **rs12413409 (CYP17A1)** |  |  |  |  |  |  |  |  |  |  |
| At low stress |  | 0.85 | 0.62 | 1.19 | n.s. |  | 0.99 | 0.80 | 1.22 | n.s. |
| At intermediate stress |  | 1.02 | 0.74 | 1.40 | n.s. |  | 0.94 | 0.77 | 1.14 | n.s. |
| At high stress |  | 1.17 | 0.61 | 2.22 | n.s. |  | 1.02 | 0.70 | 1.50 | n.s. |
|  |  |  |  |  |  |  |  |  |  |  |
| **rs964184 (APOA5)** |  |  |  |  |  |  |  |  |  |  |
| At low stress |  | 1.13 | 0.84 | 1.52 | n.s. |  | 1.05 | 0.87 | 1.26 | n.s. |
| At intermediate stress |  | 1.18 | 0.89 | 1.57 | n.s. |  | 0.89 | 0.73 | 1.08 | n.s. |
| At high stress |  | 1.06 | 0.65 | 1.75 | n.s. |  | 1.12 | 0.82 | 1.54 | n.s. |
|  |  |  |  |  |  |  |  |  |  |  |
| **rs2259816 (HNF1A)** |  |  |  |  |  |  |  |  |  |  |
| At low stress |  | 0.85 | 0.68 | 1.07 | n.s. |  | **0.85** | **0.74** | **0.98** | **0.021** |
| At intermediate stress |  | 0.93 | 0.76 | 1.14 | n.s. |  | 0.99 | 0.87 | 1.13 | n.s. |
| At high stress |  | 0.59 | 0.39 | 0.89 | n.s. |  | 1.15 | 0.91 | 1.46 | **0.030** |
|  |  |  |  |  |  |  |  |  |  |  |
| **rs3184504 (SH2B3)** |  |  |  |  |  |  |  |  |  |  |
| At low stress |  | 1.08 | 0.88 | 1.34 | n.s. |  | 0.99 | 0.87 | 1.13 | n.s. |
| At intermediate stress |  | 1.10 | 0.91 | 1.34 | n.s. |  | 1.16 | 1.02 | 1.31 | n.s. |
| At high stress |  | 1.01 | 0.71 | 1.45 | n.s. |  | 1.04 | 0.83 | 1.31 | n.s. |
|  |  |  |  |  |  |  |  |  |  |  |
| **rs4773144 (COL4A1)** |  |  |  |  |  |  |  |  |  |  |
| At low stress |  | 0.97 | 0.78 | 1.20 | n.s. |  | 0.98 | 0.86 | 1.11 | n.s. |
| At intermediate stress |  | 1.02 | 0.84 | 1.25 | n.s. |  | 1.08 | 0.95 | 1.23 | n.s. |
| At high stress |  | **0.60** | **0.40** | **0.88** | **0.036** |  | 1.13 | 0.90 | 1.42 | n.s. |
|  |  |  |  |  |  |  |  |  |  |  |
| **rs2895811 (HHIPL1)** |  |  |  |  |  |  |  |  |  |  |
| At low stress |  | **1.32** | **1.07** | **1.64** | **0.010** |  | 0.90 | 0.79 | 1.02 | n.s. |
| At intermediate stress |  | 0.93 | 0.76 | 1.13 | **0.016** |  | **1.19** | **1.05** | **1.34** | **0.002** |
| At high stress |  | 1.50 | 1.04 | 2.15 | n.s. |  | 1.07 | 0.85 | 1.35 | n.s. |
|  |  |  |  |  |  |  |  |  |  |  |
| **rs3825807 (ADAMTS7)** |  |  |  |  |  |  |  |  |  |  |
| At low stress |  | 1.05 | 0.85 | 1.30 | n.s. |  | 0.94 | 0.83 | 1.07 | n.s. |
| At intermediate stress |  | **0.82** | **0.68** | **0.99** | n.s. |  | 1.06 | 0.94 | 1.21 | n.s. |
| At high stress |  | 1.26 | 0.87 | 1.82 | n.s. |  | 0.87 | 0.69 | 1.09 | n.s. |
|  |  |  |  |  |  |  |  |  |  |  |
| **rs12936587 (RASD1)** |  |  |  |  |  |  |  |  |  |  |
| At low stress |  | 0.95 | 0.77 | 1.17 | n.s. |  | 1.10 | 0.97 | 1.25 | n.s. |
| At intermediate stress |  | 1.09 | 0.90 | 1.33 | n.s. |  | 1.01 | 0.89 | 1.15 | n.s. |
| At high stress |  | 1.29 | 0.90 | 1.85 | n.s. |  | 1.09 | 0.87 | 1.37 | n.s. |
|  |  |  |  |  |  |  |  |  |  |  |
| **rs216172 (SMG6)** |  |  |  |  |  |  |  |  |  |  |
| At low stress |  | **1.26** | **1.01** | **1.56** | **0.038** |  | 1.05 | 0.92 | 1.21 | n.s. |
| At intermediate stress |  | 0.94 | 0.76 | 1.16 | n.s. |  | 0.99 | 0.87 | 1.13 | n.s. |
| At high stress |  | 1.19 | 0.83 | 1.69 | n.s. |  | 0.95 | 0.75 | 1.20 | n.s. |
|  |  |  |  |  |  |  |  |  |  |  |
| **rs318090 (UBE2Z)** |  |  |  |  |  |  |  |  |  |  |
| At low stress |  | 0.94 | 0.76 | 1.16 | n.s. |  | 1.03 | 0.91 | 1.17 | n.s. |
| At intermediate stress |  | 1.16 | 0.95 | 1.42 | n.s. |  | **1.16** | **1.02** | **1.31** | n.s. |
| At high stress |  | 1.20 | 0.83 | 1.73 | n.s. |  | 1.16 | 0.92 | 1.46 | n.s. |
|  |  |  |  |  |  |  |  |  |  |  |
| **rs1122608 (LDLR)** |  |  |  |  |  |  |  |  |  |  |
| At low stress |  | 1.01 | 0.78 | 1.30 | n.s. |  | 0.94 | 0.81 | 1.10 | n.s. |
| At intermediate stress |  | 1.02 | 0.80 | 1.29 | n.s. |  | 1.01 | 0.87 | 1.18 | n.s. |
| At high stress |  | 1.10 | 0.72 | 1.67 | n.s. |  | 0.99 | 0.76 | 1.29 | n.s. |
|  |  |  |  |  |  |  |  |  |  |  |
| **rs9982601 (KCNE2)** |  |  |  |  |  |  |  |  |  |  |
| At low stress |  | 1.03 | 0.76 | 1.41 | n.s. |  | 1.06 | 0.88 | 1.27 | n.s. |
| At intermediate stress |  | 1.16 | 0.89 | 1.52 | n.s. |  | 1.06 | 0.89 | 1.27 | n.s. |
| At high stress |  | 1.30 | 0.81 | 2.10 | n.s. |  | 0.99 | 0.71 | 1.38 | n.s. |
|  |  |  |  |  |  |  |  |  |  |  |
| **rs4845625 (IL6R)** |  |  |  |  |  |  |  |  |  |  |
| At low stress |  | 0.95 | 0.77 | 1.18 | n.s. |  | 1.04 | 0.91 | 1.18 | n.s. |
| At intermediate stress |  | 1.09 | 0.89 | 1.33 | n.s. |  | **1.17** | **1.03** | **1.33** | n.s. |
| At high stress |  | 0.79 | 0.54 | 1.16 | n.s. |  | 1.10 | 0.87 | 1.39 | n.s. |
|  |  |  |  |  |  |  |  |  |  |  |
| **rs2028900 (GGCX/VAMP8)** |  |  |  |  |  |  |  |  |  |  |
| At low stress |  | 0.99 | 0.80 | 1.22 | n.s. |  | 1.04 | 0.91 | 1.18 | n.s. |
| At intermediate stress |  | 0.93 | 0.76 | 1.13 | n.s. |  | 0.95 | 0.84 | 1.08 | n.s. |
| At high stress |  | 1.38 | 0.96 | 1.98 | n.s. |  | 0.99 | 0.79 | 1.25 | n.s. |
|  |  |  |  |  |  |  |  |  |  |  |
| **rs4299376 (ABCG8)** |  |  |  |  |  |  |  |  |  |  |
| At low stress |  | 1.28 | 1.00 | 1.64 | n.s. |  | 0.96 | 0.83 | 1.10 | n.s. |
| At intermediate stress |  | 1.03 | 0.83 | 1.28 | n.s. |  | 0.92 | 0.81 | 1.05 | n.s. |
| At high stress |  | 0.82 | 0.56 | 1.21 | n.s. |  | 1.14 | 0.88 | 1.48 | n.s. |
|  |  |  |  |  |  |  |  |  |  |  |
| **rs515135 (APOB)** |  |  |  |  |  |  |  |  |  |  |
| At low stress |  | 0.92 | 0.70 | 1.21 | n.s. |  | 1.04 | 0.87 | 1.23 | n.s. |
| At intermediate stress |  | 0.96 | 0.74 | 1.24 | n.s. |  | 1.11 | 0.93 | 1.32 | n.s. |
| At high stress |  | 0.99 | 0.60 | 1.65 | n.s. |  | 0.84 | 0.62 | 1.13 | n.s. |
|  |  |  |  |  |  |  |  |  |  |  |
| **Table A2 continued** |  |  |  |  |  |  |  |  |  |  |
|  |  |  |  |  |  |  |  |  |  |  |
| **rs2252641 (ZEB2-AC074093.1)** |  |  |  |  |  |  |  |  |  |  |
| At low stress |  | 1.20 | 0.97 | 1.50 | n.s. |  | 1.08 | 0.95 | 1.23 | n.s. |
| At intermediate stress |  | 1.05 | 0.87 | 1.28 | n.s. |  | 1.05 | 0.93 | 1.19 | n.s. |
| At high stress |  | 0.91 | 0.64 | 1.31 | n.s. |  | **0.76** | **0.60** | **0.96** | **0.010** |
|  |  |  |  |  |  |  |  |  |  |  |
| **rs1878406 (EDNRA)** |  |  |  |  |  |  |  |  |  |  |
| At low stress |  | 1.29 | 0.96 | 1.73 | n.s. |  | 1.09 | 0.90 | 1.31 | n.s. |
| At intermediate stress |  | 0.81 | 0.59 | 1.12 | **0.039** |  | 1.10 | 0.91 | 1.32 | n.s. |
| At high stress |  | 0.87 | 0.48 | 1.55 | n.s. |  | 1.04 | 0.74 | 1.46 | n.s. |
|  |  |  |  |  |  |  |  |  |  |  |
| **rs7692387 (GUCY1A3)** |  |  |  |  |  |  |  |  |  |  |
| At low stress |  | 1.00 | 0.76 | 1.31 | n.s. |  | 1.05 | 0.89 | 1.24 | n.s. |
| At intermediate stress |  | 1.00 | 0.79 | 1.28 | n.s. |  | 1.09 | 0.93 | 1.27 | n.s. |
| At high stress |  | 0.99 | 0.62 | 1.56 | n.s. |  | 0.96 | 0.72 | 1.28 | n.s. |
|  |  |  |  |  |  |  |  |  |  |  |
| **rs273909 (SLC22A4/SLC22A5)** |  |  |  |  |  |  |  |  |  |  |
| At low stress |  | 1.15 | 0.85 | 1.55 | n.s. |  | 0.94 | 0.78 | 1.13 | n.s. |
| At intermediate stress |  | 1.11 | 0.83 | 1.48 | n.s. |  | 0.98 | 0.81 | 1.19 | n.s. |
| At high stress |  | 0.82 | 0.45 | 1.47 | n.s. |  | 0.78 | 0.54 | 1.14 | n.s. |
|  |  |  |  |  |  |  |  |  |  |  |
| **rs10947789 (KCNK5)** |  |  |  |  |  |  |  |  |  |  |
| At low stress |  | 1.04 | 0.81 | 1.33 | n.s. |  | 1.11 | 0.95 | 1.29 | n.s. |
| At intermediate stress |  | 0.91 | 0.73 | 1.12 | n.s. |  | 0.99 | 0.86 | 1.15 | n.s. |
| At high stress |  | 1.16 | 0.76 | 1.76 | n.s. |  | 1.15 | 0.88 | 1.51 | n.s. |
|  |  |  |  |  |  |  |  |  |  |  |
| **rs2048327 (SLC22A3/LPAL2/LPA)** |  |  |  |  |  |  |  |  |  |  |
| At low stress |  | 1.11 | 0.90 | 1.37 | n.s. |  | 1.03 | 0.90 | 1.17 | n.s. |
| At intermediate stress |  | 1.05 | 0.86 | 1.28 | n.s. |  | 1.05 | 0.92 | 1.19 | n.s. |
| At high stress |  | 0.86 | 0.60 | 1.25 | n.s. |  | 0.93 | 0.74 | 1.18 | n.s. |
|  |  |  |  |  |  |  |  |  |  |  |
| **rs4252120 (PLG)** |  |  |  |  |  |  |  |  |  |  |
| At low stress |  | 1.02 | 0.81 | 1.29 | n.s. |  | 1.07 | 0.93 | 1.24 | n.s. |
| At intermediate stress |  | 1.07 | 0.85 | 1.33 | n.s. |  | 1.03 | 0.90 | 1.19 | n.s. |
| At high stress |  | 1.35 | 0.90 | 2.03 | n.s. |  | 1.28 | 0.98 | 1.66 | n.s. |
|  |  |  |  |  |  |  |  |  |  |  |
| **rs11984041 (HDAC9)** |  |  |  |  |  |  |  |  |  |  |
| At low stress |  | 1.37 | 0.98 | 1.93 | n.s. |  | 1.19 | 0.96 | 1.47 | n.s. |
| At intermediate stress |  | 1.05 | 0.74 | 1.48 | n.s. |  | 1.07 | 0.86 | 1.32 | n.s. |
| At high stress |  | 1.55 | 0.90 | 2.67 | n.s. |  | 1.12 | 0.76 | 1.65 | n.s. |
|  |  |  |  |  |  |  |  |  |  |  |
| **rs10953541 (BCAP29)** |  |  |  |  |  |  |  |  |  |  |
| At low stress |  | 0.98 | 0.76 | 1.25 | n.s. |  | 1.15 | 0.99 | 1.35 | n.s. |
| At intermediate stress |  | 0.94 | 0.75 | 1.17 | n.s. |  | 0.94 | 0.82 | 1.09 | n.s. |
| At high stress |  | 1.17 | 0.76 | 1.78 | n.s. |  | 0.98 | 0.76 | 1.27 | n.s. |
|  |  |  |  |  |  |  |  |  |  |  |
| **rs2954029 (TRIB1)** |  |  |  |  |  |  |  |  |  |  |
| At low stress |  | 0.88 | 0.71 | 1.08 | n.s. |  | 1.08 | 0.95 | 1.23 | n.s. |
| At intermediate stress |  | 0.99 | 0.82 | 1.21 | n.s. |  | 0.99 | 0.87 | 1.12 | n.s. |
| At high stress |  | **1.57** | **1.08** | **2.28** | **0.008** |  | 1.08 | 0.86 | 1.36 | n.s. |
|  |  |  |  |  |  |  |  |  |  |  |
| **rs3217992 (CDKN2BAS)** |  |  |  |  |  |  |  |  |  |  |
| At low stress |  | **1.25** | **1.01** | **1.55** | **0.043** |  | **1.17** | **1.02** | **1.33** | **0.02** |
| At intermediate stress |  | 1.13 | 0.92 | 1.38 | n.s. |  | 0.97 | 0.85 | 1.11 | n.s. |
| At high stress |  | 0.92 | 0.63 | 1.36 | n.s. |  | 1.46 | 1.16 | 1.84 | n.s. |
|  |  |  |  |  |  |  |  |  |  |  |
| **rs2487928 (KIAA1462)** |  |  |  |  |  |  |  |  |  |  |
| At low stress |  | 0.88 | 0.71 | 1.10 | n.s. |  | 1.04 | 0.91 | 1.18 | n.s. |
| At intermediate stress |  | 0.72 | 0.59 | 0.87 | n.s. |  | 1.06 | 0.93 | 1.20 | n.s. |
| At high stress |  | 0.92 | 0.65 | 1.30 | n.s. |  | 1.04 | 0.83 | 1.31 | n.s. |
|  |  |  |  |  |  |  |  |  |  |  |
| **rs2047009 (CXCL12)** |  |  |  |  |  |  |  |  |  |  |
| At low stress |  | 1.09 | 0.88 | 1.35 | n.s. |  | 1.08 | 0.95 | 1.22 | n.s. |
| At intermediate stress |  | 1.00 | 0.82 | 1.22 | n.s. |  | 1.13 | 0.99 | 1.28 | n.s. |
| At high stress |  | 0.89 | 0.62 | 1.28 | n.s. |  | **1.34** | **1.06** | **1.69** | n.s. |
|  |  |  |  |  |  |  |  |  |  |  |
| **rs1412444 (LIPA)** |  |  |  |  |  |  |  |  |  |  |
| At low stress |  | 1.04 | 0.82 | 1.31 | n.s. |  | 1.09 | 0.95 | 1.25 | n.s. |
| At intermediate stress |  | 1.07 | 0.87 | 1.33 | n.s. |  | 1.05 | 0.92 | 1.20 | n.s. |
| At high stress |  | 1.18 | 0.81 | 1.70 | n.s. |  | 0.93 | 0.73 | 1.19 | n.s. |
|  |  |  |  |  |  |  |  |  |  |  |
| **rs11226029 (PDGFD)** |  |  |  |  |  |  |  |  |  |  |
| At low stress |  | 1.02 | 0.80 | 1.30 | n.s. |  | 0.89 | 0.77 | 1.03 | n.s. |
| At intermediate stress |  | 1.11 | 0.88 | 1.39 | n.s. |  | 0.97 | 0.84 | 1.11 | n.s. |
| At high stress |  | 0.89 | 0.59 | 1.34 | n.s. |  | **1.45** | **1.08** | **1.94** | **0.003** |
|  |  |  |  |  |  |  |  |  |  |  |
| **rs9319428 (FLT1)** |  |  |  |  |  |  |  |  |  |  |
| At low stress |  | 1.01 | 0.80 | 1.26 | n.s. |  | 0.88 | 0.77 | 1.02 | n.s. |
| At intermediate stress |  | 1.24 | 1.01 | 1.52 | n.s. |  | 1.06 | 0.93 | 1.21 | n.s. |
| At high stress |  | 1.20 | 0.83 | 1.74 | n.s. |  | 1.21 | 0.95 | 1.53 | **0.028** |
|  |  |  |  |  |  |  |  |  |  |  |
| **Table A2 continued** |  |  |  |  |  |  |  |  |  |  |
|  |  |  |  |  |  |  |  |  |  |  |
| **rs9515203 (COL4A1/COL4A2)** |  |  |  |  |  |  |  |  |  |  |
| At low stress |  | 1.10 | 0.86 | 1.40 | n.s. |  | 1.07 | 0.93 | 1.24 | n.s. |
| At intermediate stress |  | 1.04 | 0.84 | 1.29 | n.s. |  | **1.18** | **1.03** | **1.37** | n.s. |
| At high stress |  | 0.83 | 0.57 | 1.19 | n.s. |  | 1.12 | 0.87 | 1.44 | n.s. |
|  |  |  |  |  |  |  |  |  |  |  |
| **rs7173743 (ADAMTS7)** |  |  |  |  |  |  |  |  |  |  |
| At low stress |  | 1.15 | 0.93 | 1.44 | n.s. |  | 1.03 | 0.90 | 1.17 | n.s. |
| At intermediate stress |  | 0.87 | 0.72 | 1.06 | n.s. |  | 1.08 | 0.95 | 1.22 | n.s. |
| At high stress |  | 1.67 | 1.13 | 2.48 | n.s. |  | 0.92 | 0.73 | 1.16 | n.s. |
|  |  |  |  |  |  |  |  |  |  |  |
| **rs17514846 (FURIN/FES)** |  |  |  |  |  |  |  |  |  |  |
| At low stress |  | 1.04 | 0.84 | 1.29 | n.s. |  | 1.12 | 0.98 | 1.28 | n.s. |
| At intermediate stress |  | 1.09 | 0.90 | 1.32 | n.s. |  | 1.00 | 0.88 | 1.13 | n.s. |
| At high stress |  | 1.00 | 0.69 | 1.44 | n.s. |  | 0.95 | 0.75 | 1.20 | n.s. |

**_______________________***P-values are for the interaction between SNP and stress level.

CAD=Coronary Artery Disease; CI=Confidence Interval; MI=Myocardial Infarction; n.s.=non-significant; SNP=Single nucleotide polymorphism;

Analyses are adjusted for gender, age, education, socioeconomic index, smoking, drinking, prevalent diabetes mellitus, Body Mass Index, hypertension, and use of lipid lowering medication

Table B. Cox proportional hazards multivariable models for the interaction between psychological stress and a stress-sensitive component of the genetic risk score (GRS) for coronary artery disease (CAD), fatal myocardial infarction (MI) and cardiovascular death

|  |  | Stress-sensitive GRS Quartiles | | | | | | |  | |
| --- | --- | --- | --- | --- | --- | --- | --- | --- | --- | --- |
| End point |  | Q1 |  | Q2 |  | Q3 |  | Q4 |  | P for interaction^*^ |
| **CAD** |  |  |  |  |  |  |  |  |  |  |
| No. (Events) |  | 3270 (316) |  | 5623 (586) |  | 5784 (591) |  | 3882 (445) |  |  |
|  |  | HR (95% CI) |  | HR (95% CI) |  | HR (95% CI) |  | HR (95% CI) |  |  |
| Multivariable model^†^ |  |  |  |  |  |  |  |  |  |  |
| Low stress |  | Reference |  | 1.12 (0.91-1.39) |  | 1.11 (0.89-1.37) |  | **1.34* (1.07-1.67)** |  |  |
| Intermediate stress |  | 1.07 (0.84-1.36) |  | 1.13 (0.91-1.40) |  | 1.14 (0.92-1.41) |  | 1.13 (0.90-1.42) |  |  |
| High stress |  | 0.99 (0.69-1.40) |  | 1.15 (0.87-1.53) |  | 0.96 (0.71-1.29) |  | **1.47* (1.09-1.99)** |  | n.s. |
|  |  |  |  |  |  |  |  |  |  |  |
|  |  |  |  |  |  |  |  |  |  |  |
| **Fatal MI** |  |  |  |  |  |  |  |  |  |  |
| No. (Events) |  | 3270 (69) |  | 5623 (133) |  | 5784 (136) |  | 3882 (98) |  |  |
|  |  | HR (95% CI) |  | HR (95% CI) |  | HR (95% CI) |  | HR (95% CI) |  |  |
| Multivariable model^†^ |  |  |  |  |  |  |  |  |  |  |
| Low stress |  | Reference |  | 1.36 (0.84-2.20) |  | 1.30 (0.80-2.11) |  | 1.31 (0.77-2.21) |  |  |
| Intermediate stress |  | 1.48 (0.88-2.47) |  | 1.41 (0.87-2.29) |  | 1.44 (0.89-2.31) |  | 1.15 (0.68-1.93) |  |  |
| High stress |  | 0.78 (0.32-1.92) |  | 1.40 (0.73-2.66) |  | 1.12 (0.58-2.19) |  | **3.19*** (1.79-5.68)** |  | **0.009** |
|  |  |  |  |  |  |  |  |  |  |  |
| **Cardiovascular death** |  |  |  |  |  |  |  |  |  |  |
| No. (Events) |  | 3270 (191) |  | 5623 (318) |  | 5784 (340) |  | 3882 (222) |  |  |
|  |  | HR (95% CI) |  | HR (95% CI) |  | HR (95% CI) |  | HR (95% CI) |  |  |
| Multivariable model^†^ |  |  |  |  |  |  |  |  |  |  |
| Low stress |  | Reference |  | 0.83 (0.64-1.09) |  | 0.85 (0.65-1.11) |  | 0.77 (0.57-1.03) |  |  |
| Intermediate stress |  | 0.86 (0.64-1.15) |  | 0.87 (0.66-1.13) |  | 0.93 (0.72-1.21) |  | 0.79 (0.59-1.05) |  |  |
| High stress |  | **0.43** (0.23-0.78)** |  | 0.94 (0.65-1.37) |  | 0.87 (0.60-1.27) |  | 1.35 (0.92-1.98) |  | **0.004** |

*p<0.05; **p<0.01, ***p<0.001;

CAD=Coronary Artery Disease; CI=Confidence Interval; GRS=Genetic risk score; HR=Hazard Ratio; MI=Myocardial Infarction; n.s.=non-significant;

^†^Multivariable models are adjusted for gender, age, education, socioeconomic index, smoking, drinking, prevalent diabetes mellitus, Body Mass Index, hypertension, and use of lipid lowering medication

Table C. Hazard Ratios (HR) and Confidence Intervals (CI) for the main effects of a lipoprotein genetic risk score (GRS) and stress on incidence of coronary artery disease (CAD)

|  |  | Lipoprotein-GRS Quartiles | | | |  |  | Stress | | |  |
| --- | --- | --- | --- | --- | --- | --- | --- | --- | --- | --- | --- |
|  |  | Q1 | Q2 | Q3 | Q4 | P for trend |  | Low | Intermediate | High | P for trend |
| **CAD** |  |  |  |  |  |  |  |  |  |  |  |
| No. (Events) |  | 3818 (366) | 6737 (662) | 1672 (191) | 6332 (719) |  |  | 7234 (817) | 8227 (864) | 3098 (257) |  |
| Minimal model^*^ HR  (95% CI) |  | Reference | 1.00  (0.88-1.14) | **1.21  (1.02-1.45)** | **1.20  (1.06-1.36)** |  |  | Reference | 1.08  (0.98-1.19) | 1.11  (0.96-1.28) |  |
| P value |  | - | n.s. | 0.03 | 0.005 |  |  | - | n.s. | n.s. |  |
|  |  |  |  |  |  |  |  |  |  |  |  |
| Multivariable model^†^ HR  (95% CI) |  | Reference | 1.01  (0.89-1.15) | **1.21  (1.02-1.45)** | **1.18  (1.04-1.34)** | **0.001** |  | Reference | 0.98  (0.89-1.09) | 0.99  (0.85-1.14) | n.s. |
| P value |  | - | n.s. | 0.03 | 0.01 |  |  | - | n.s. | n.s. |  |

CAD=Coronary Artery Disease; CI=Confidence Interval; GRS=Genetic risk score; HR=Hazard Ratio; n.s.=non-significant;

^*^Minimal models are adjusted for gender and age

^†^Multivariable models are adjusted for gender, age, education, socioeconomic index, smoking, drinking, prevalent diabetes mellitus, Body Mass Index, hypertension, and use of lipid lowering medication

Table D. Hazard Ratios (HR) and Confidence Intervals (CI) for the main effects of a lipid genetic risk score (GRS) and stress on incidence of coronary artery disease (CAD)

|  |  | Lipid-GRS Quartiles | | | |  |  | Stress | | |  |
| --- | --- | --- | --- | --- | --- | --- | --- | --- | --- | --- | --- |
|  |  | Q1 | Q2 | Q3 | Q4 | P for trend |  | Low | Intermediate | High | P for trend |
| **CAD** |  |  |  |  |  |  |  |  |  |  |  |
| No. (Events) |  | 4750 (472) | 4607 (495) | 2981 (296) | 6221 (675) |  |  | 7234 (817) | 8227 (864) | 3098 (257) |  |
| Minimal model^*^ HR  (95% CI) |  | Reference | 1.10  (0.97-1.25) | 0.99  (0.85-1.14) | **1.14  (1.01-1.28)** |  |  | Reference | 1.08  (0.98-1.19) | 1.11  (0.96-1.27) |  |
| P value |  | - | n.s. | n.s. | 0.03 |  |  | - | n.s. | n.s. |  |
|  |  |  |  |  |  |  |  |  |  |  |  |
| Multivariable model^†^ HR  (95% CI) |  | Reference | 1.10  (0.97-1.25) | 0.96  (0.83-1.11) | **1.14  (1.01-1.28)** | n.s. |  | Reference | 0.98  (0.89-1.08) | 0.98  (0.85-1.13) | n.s. |
| P value |  | - | n.s. | n.s. | 0.03 |  |  | - | n.s. | n.s. |  |

CAD=Coronary Artery Disease; CI=Confidence Interval; GRS=Genetic risk score; HR=Hazard Ratio; n.s.=non-significant;

^*^Minimal models are adjusted for gender and age

^†^Multivariable models are adjusted for gender, age, education, socioeconomic index, smoking, drinking, prevalent diabetes mellitus, Body Mass Index, hypertension, and use of lipid lowering medication

Table E. Hazard Ratios (HR) and Confidence Intervals (CI) for the main effects of a inflammation genetic risk score (GRS) and stress on incidence of coronary artery disease (CAD), fatal myocardial infarction (MI), non-fatal MI, and cardiovascular death

|  |  | Inflammation-GRS Quartiles | | | |  |  | Stress | | |  |
| --- | --- | --- | --- | --- | --- | --- | --- | --- | --- | --- | --- |
|  |  | Q1 | Q2 | Q3 | Q4 | P for trend |  | Low | Intermediate | High | P for trend |
| **CAD** |  |  |  |  |  |  |  |  |  |  |  |
| No. (Events) |  | 4198 (406) | 3449 (334) | 5326 (549) | 5586 (649) |  |  | 7234 (817) | 8227 (864) | 3098 (257) |  |
| Minimal model^*^ HR  (95% CI) |  | Reference | 1.02  (0.88-1.18) | 1.07  (0.94-1.22) | **1.21  (1.07-1.37)** |  |  | Reference | 1.08  (0.98-1.18) | 1.11  (0.96-1.28) |  |
| P value |  | - | n.s. | n.s. | 0.002 |  |  | - | n.s. | n.s. |  |
|  |  |  |  |  |  |  |  |  |  |  |  |
| Multivariable model^†^ HR  (95% CI) |  | Reference | 1.03  (0.89-1.20) | 1.10  (0.97-1.25) | **1.23  (1.08-1.39)** | **<0.001** |  | Reference | 0.98  (0.89-1.08) | 0.99  (0.85-1.14) | n.s. |
| P value |  | - | n.s. | n.s. | 0.001 |  |  | - | n.s. | n.s. |  |
|  |  |  |  |  |  |  |  |  |  |  |  |
|  |  |  |  |  |  |  |  |  |  |  |  |
| **Fatal MI** |  |  |  |  |  |  |  |  |  |  |  |
| No. (Events) |  | 4198 (87) | 3449 (79) | 5326 (122) | 5586 (148) |  |  | 7234 (173) | 8227 (203) | 3098 (60) |  |
| Minimal model^*^ HR  (95% CI) |  | Reference | 1.13  (0.83-1.53) | 1.11  (0.85-1.47) | 1.28  (0.99-1.67) |  |  | Reference | **1.23**  **(1.00-1.51)** | **1.42**  **(1.06-1.92)** |  |
| P value |  | - | n.s. | n.s. | n.s. |  |  | - | 0.047 | 0.021 |  |
|  |  |  |  |  |  |  |  |  |  |  |  |
| Multivariable model^†^ HR  (95% CI) |  | Reference | 1.13  (0.83-1.54) | 1.15  (0.87-1.51) | **1.31**  **(1.00-1.71)** | n.s. |  | Reference | 1.08  (0.88-1.33) | 1.21  (0.90-1.64) | n.s. |
| P value |  | - | n.s. | n.s. | 0.048 |  |  | - | n.s. | n.s. |  |
|  |  |  |  |  |  |  |  |  |  |  |  |
|  |  |  |  |  |  |  |  |  |  |  |  |
| **Non-fatal MI** |  |  |  |  |  |  |  |  |  |  |  |
| No. (Events) |  | 4198 (232) | 3449 (189) | 5326 (320) | 5586 (367) |  |  | 7234 (470) | 8227 (490) | 3098 (148) |  |
| Minimal model^*^ HR  (95% CI) |  | Reference | 1.01  (0.83-1.22) | 1.10  (0.92-1.30) | **1.20**  **(1.02-1.41)** |  |  | Reference | 1.04  (0.92-1.18) | 1.07  (0.89-1.29) |  |
| P value |  | - | n.s. | n.s. | 0.03 |  |  | - | n.s. | n.s. |  |
|  |  |  |  |  |  |  |  |  |  |  |  |
| Multivariable model^†^ HR  (95% CI) |  | Reference | 1.02  (0.85-1.24) | 1.12  (0.95-1.33) | **1.21**  **(1.03-1.43)** | **0.01** |  | Reference | 0.96  (0.84-1.09) | 0.97  (0.80-1.17) | n.s. |
| P value |  | - | n.s. | n.s. | 0.02 |  |  | - | n.s. | n.s. |  |
|  |  |  |  |  |  |  |  |  |  |  |  |
|  |  |  |  |  |  |  |  |  |  |  |  |
|  |  |  |  |  |  |  |  |  |  |  |  |
|  |  |  |  |  |  |  |  |  |  |  |  |
|  |  |  |  |  |  |  |  |  |  |  |  |
|  |  |  |  |  |  |  |  |  |  |  |  |
| **Table E continued** |  |  |  |  |  |  |  |  |  |  |  |
|  |  |  |  |  |  |  |  |  |  |  |  |
| **Cardiovascular death** |  |  |  |  |  |  |  |  |  |  |  |
| No. (Events) |  | 4198 (231) | 3449 (181) | 5326 (297) | 5586 (362) |  |  | 7234 (448) | 8227 (493) | 3098 (130) |  |
| Minimal model^*^ HR  (95% CI) |  | Reference | 0.98  (0.81-1.19) | 1.03  (0.86-1.22) | **1.19**  **(1.01-1.40)** |  |  | Reference | 1.12  (0.99-1.28) | 1.21  (0.99-1.47) |  |
| P value |  | - | n.s. | n.s. | 0.04 |  |  | - | n.s. | n.s. |  |
|  |  |  |  |  |  |  |  |  |  |  |  |
| Multivariable model^†^ HR  (95% CI) |  | Reference | 0.98  (0.81-1.19) | 1.05  (0.88-1.24) | **1.22**  **(1.03-1.44)** | **0.01** |  | Reference | 1.02  (0.89-1.16) | 1.05  (0.86-1.29) | n.s. |
| P value |  | - | n.s. | n.s. | 0.02 |  |  | - | n.s. | n.s. |  |

CAD=Coronary Artery Disease; CI=Confidence Interval; GRS=Genetic risk score; HR=Hazard Ratio; n.s.=non-significant;

^*^Minimal models are adjusted for gender and age

^†^Multivariable models are adjusted for gender, age, education, socioeconomic index, smoking, drinking, prevalent diabetes mellitus, Body Mass Index, hypertension, and use of lipid lowering medication
